# Assessment of soil amplification effects on the seismic vulnerability of irregular reinforced concrete buildings of varying heights

**DOI:** 10.1038/s41598-025-14145-2

**Published:** 2025-09-30

**Authors:** Aman Kumar, Goutam Ghosh

**Affiliations:** Civil Engineering Department, Motilal Nehru National Institute of Technology Allahabad, Prayagraj, 211004 UP India

**Keywords:** Soil amplification, Pushover analysis, Unsymmetrical RC building, Building height, Time history analysis, Bed rock level, Surface level, Probability of damage, Damage index, Recovery time, And seismic vulnerability index, Natural hazards, Engineering

## Abstract

Earthquakes pose a significant threat to structures in seismically active regions. It is, therefore, important to understand the factors that influence the vulnerability of buildings. The seismic performance of buildings is significantly influenced by soil amplification, which depends upon the soil type and ground motion characteristics. In addition, building height and geometric configuration, especially for asymmetric structures such as L-shaped buildings, play a crucial role due to the different stiffness, flexibility, and torsional effects. This present study investigates the impact of soil amplification and ground motion characteristics on the seismic vulnerability of unsymmetrical L-shaped buildings of varying heights by considering five different soil profiles (3 homogeneous and 2 layered) and five different ground motions (two far-field and three near-field). Reinforced concrete moment-resisting frame buildings exhibit significant nonlinear behavior under strong seismic excitation, which must be accurately captured to assess their seismic performance. In this study, the pushover and time history analysis have been performed to estimate the seismic response of the building in terms of base shear, roof displacement, and demand ductility. Finally, fragility analysis has been conducted to estimate the probability of damage, damage index, seismic vulnerability index, and recovery time of the building considering the amplified ground motion effects caused by various soil profiles. The analysis reveals that near-field ground motions significantly amplify ground motion which results in increased roof displacement values (up to 211%), collapse damage (up to 37%), damage index (up to 162%), seismic vulnerability index (up to 189%) and recovery time (up to 383 days) of the building compared to far-field motions. Soil amplification effects are most significant in low-rise buildings, while with increasing building height of unsymmetrical, vulnerability rises due to torsional effects. Layered soil profiles in Silchar and Turkey increase the vulnerability of low-rise buildings, whereas homogeneous clay soil poses greater risks for high-rise buildings. This study highlights the need for Indian Standard (IS) codes to incorporate soil-specific amplification factors and building height considerations, offering practical recommendations to improve seismic design practices for unsymmetrical buildings.

## Introduction

The seismic vulnerability of the buildings to seismic activity has consistently gathered the attention of civil and structural engineers because of the devastating nature of earthquakes. Historically, failures in asymmetrical buildings during earthquakes have underscored the significance of current design methodologies and a complete understanding of their performance under seismic forces. Several older buildings do not meet present seismic standards, highlighting the need for evaluations to verify their capacity to endure possible earthquakes. Seismic vulnerability assessment is crucial in areas with both historical and modern buildings. It helps to identify at-risk structures, enabling targeted mitigation strategies to reduce economic losses and enhance earthquake resilience. (Granados et al., 2024^1^; EI-Betar, 2018^2^; Bendehiba et al., 2023^3^; Krzan & Bosiljkov, 2023^4^.

Seismic vulnerability of buildings is influenced by ground motion characteristics, geotechnical conditions, and structural properties, as documented in the literature (Kramer, 1996^5^; ASCE, 2017^6^. This study focuses on these engineering parameters, excluding social factors. Ground motion characteristics, including peak ground acceleration (PGA), spectral acceleration, and epicentral distance, are evaluated using near-field and far-field records, which affect structural response due to variations in frequency content (Boore & Atkinson, 2008^7^; Campbell & Bozorgnia, 2008^8^. Geotechnical conditions, such as soil profiles and shear wave velocity, govern ground motion amplification (Borcherdt, 1994^9^; Hashash et al., 2010^10^. Structural properties, including building height and plan asymmetry, influence dynamic response and are assessed per seismic standards (ASCE, 2017^6^; Chopra, 2012^11^. The seismic vulnerability of the building is significantly affected by the unsymmetry of the building. Among various structural designs, L-shaped reinforced concrete (RC) frame buildings building leads to stress concentration and increased lateral forces during seismic events primarily due to the presence of re-entrant corners and torsional irregularities, which make structures more susceptible to damage (Wahane et al., 2024^12^; Raheem et al., 2018^13^; Ahmed et al., 2016^14^.This increased vulnerability requires careful design strategies to improve seismic resilience and reduce potential damage during earthquakes (Ahmed et al., 2016)^14^. Similarly, High-rise buildings are more susceptible to seismic vulnerability due to increased height, which amplifies the effects of seismic forces. Multiple studies have demonstrated that as the number of stories in a building increases, bending moments and torsional forces in beams and columns near re-entrant corners intensify, leading to elevated stress concentrations and potential early failure of structural components during earthquakes ( Jeong et al., 2020^15^; Chowdhury & Shuvo, 2024^16^; Karayannis & Naoum, 2018^17^; Aziminejad & Moghadam, 2009^18^; Jeong & Elnashai, 2005^19^; Bensalah et al., 2012^20^. These effects arise from asymmetric seismic responses in buildings with irregular plan configurations, such as re-entrant corners, which exacerbate torsional irregularity and increase seismic demands on structural elements. Research shows that taller buildings tend to undergo greater torsional moments at both the base and throughout their height, leading to heightened inter-story drifts and shear forces (Shaikh & Shakeeb, 2013^21^. As building height increases, the contribution of higher vibration modes to dynamic response intensifies, resulting in seismic demands that may exceed predictions of standard seismic design codes (Chopra, 2012^11^; Ijmulwar, & Patro, 2024^22^. Additionally, non-symmetrical buildings with torsional irregularity, caused by asymmetric mass or stiffness distributions, experience amplified seismic responses due to torsional coupling, leading to increased structural and non-structural damage (Christopoulos, 2022^23^; Ozer, 2024^24^; Anagnostopoulos et al., 2015^25^. These effects are particularly pronounced in buildings subjected to seismic pounding or irregular plan configurations, exacerbating torsional demands (Ozer, 2024^24^. To mitigate these vulnerabilities, innovative technologies such as base isolation and fluid viscous dampers are employed, either individually or in combination, to reduce seismic forces and control torsional responses in reinforced concrete structures (Ozer & Inel, 2025^26^; Kelly, 1997^27^. Advanced analytical methods, including nonlinear dynamic analysis, and performance-based design approaches further enhance the seismic resilience of such buildings (ASCE, 2017^6^; PEER, 2017^28^. The seismic performance of structures also depends on ductility and plan eccentricity. Low ductility limits energy dissipation, increasing brittle failure risk and damage probabilities in fragility curves (Fardis, 2009^29^. High ductility enhances resilience through inelastic deformation. Plan eccentricity (5–20%) intensifies torsional effects, causing uneven force distribution and greater damage in flexible directions (De-la-Colina et al., 2013^30^. Fragility curves show higher vulnerability for low-ductility, high-eccentricity structures, with steeper curves indicating severe damage risk, while high-ductility, low-eccentricity structures exhibit flatter curves and better performance.

The characteristics of ground motions, particularly near-field and far fields, significantly influence the seismic vulnerability of buildings, bridges, and other infrastructure. Near-field ground motions, recorded close to an earthquake’s epicenter, exhibit strong shaking, a broader range of frequencies, and distinct characteristics. These characteristics can lead to more severe structural damage compared to far-field ground motions, which are recorded at greater distances and generally have lower intensity and different frequency content (Li et al., 2024^31^; Bilgin, 2024^32^. Far-field ground motions with long-period characteristics can lead to considerable responses in structures that possess long natural periods, such as high-rise buildings and bridges. The low-frequency content present in far-field motions can result in significant displacements and shear strains within these structures, mainly when situated in soft soil conditions (Bilgin, 2024)^32^. The characteristics of the earthquake, such as duration and fault type, also interact with building height to influence seismic vulnerability. Taller structures experience more significant impacts from long-duration earthquakes, which can induce resonance effects within the building (Moghadam Basefat et al.,2024)^33^. Near-fault earthquakes, which are characterized by high peak ground velocities, further increase the seismic demands on taller buildings (Kumar et al., 2023)^34^.

Seismic vulnerability of structures is significantly influenced by soil amplification effects, ground motion characteristics, structural foundation type, and the presence of adjacent buildings. Soil amplification, driven by local site conditions such as soil stiffness and nonlinearity, intensifies seismic waves, increasing structural stress (Wang & Leung, 2024^35^; Labar et al., 2024^36^. Ground motion characteristics, including peak acceleration and frequency content, further dictate the seismic forces a structure endures, amplifying damage potential. The type of foundation—shallow, deep, or pile-based—directly affects how these forces are transmitted to the superstructure, with studies like Ansari et al. (2020)^37^ showing that foundation stiffness alters seismic performance. Additionally, the presence of adjacent buildings introduces structure-soil-structure interaction (SSSI), which can modify dynamic responses through wave interference (Wang et al., 2025^38^. Wang & Yang (2022)^39^ also highlighted how nonlinear soil-structure interaction exacerbates reinforced concrete frame response, while Cavalieri et al. (2020)^40^ noted its impact on pile-founded masonry buildings. Various researchers have studied the effect of soil amplification on the seismic performance of the building, such as Alothman et al. (2023)^41^ assessed RC buildings in Australia across different site classes defined by the Australian Code and Eurocode 8, showing increased seismic demand on softer soils. Navdar et al. (2024)^42^ performed nonlinear dynamic analysis with a parametric range of soil stiffnesses, demonstrating that SSI and varying soil conditions significantly influence structural response. Athmani and Ademović (2023)^43^ evaluated unreinforced masonry buildings in 15 Algerian cities using three soil classes (S1 to S3) defined by the Algerian Seismic Design Code (RPA99 code). Their results show that soft soil (S3) marginally increases seismic vulnerability compared to stiff soil (S2), while having no effect similar to rock (S1), underscoring the importance of soil classification in vulnerability assessments. Fiamingo et al. (2022)^44^ analyzed a Mt. Etna earthquake-damaged structure and emphasized the importance of incorporating site response due to local volcanic soil characteristics, which are often neglected in design codes. Yön and Calayir (2015)^45^ studied RC buildings on Turkish code-defined soil classes (Z1–Z4), finding that very soft soils (Z4) led to the highest inter-story drifts and damage, confirming the vulnerability associated with soft soil conditions.

Timilsina et al. (2023)^46^ conducted a SAP2000-based SSI analysis of 3–10 storey RC-framed buildings on various soils (depth ~ 30 m). They explicitly modeled “very soft soil” (soil type D, Vs < 150 m/s), finding a 23% period lengthening and up to 282% increase in lateral deflection for Kobe earthquake input on very soft soil. Yang et al. (2024)^47^ analyzed an RC frame–shear wall building under long-period seismic input and found that loess (clayey soil) increased displacement amplification notably more than sand, while base shear decreased due to SSI. Similarly, Verma & Debbarma (2023)^48^ used spring-element modeling for an RC G + 10 shear-wall structure on soft, medium, and hard clayey soils, reporting marked reductions in base shear and increases in drift on soft clay, underscoring the crucial role of cohesive soils in realistic SSI assessments. Ozmen (2023)^49^ investigated soil amplification using over 8,000 simulations across 100 soil profiles and 84 ground motions. The study found that seismic code site coefficients tend to overestimate amplification on soft soils and underestimate it on stiff soils. It emphasized the need for site-specific analysis, especially for ZF-class (very soft) soils. It emphasized the need for site-specific analysis, especially for ZF-class (very soft) soils. Silvestri et al. (2024)^50^ found that SSI on shallow foundations—especially over soft soils—markedly increases building period, damping, and overturning risk. Chatterjee & Sengupta (2024)^51^ similarly demonstrated that soil-foundation flexibility significantly alters base shear and story displacement in asymmetric RC structures. Dhakal & Chaulagain (2023)^52^ explicitly compared seismic responses of irregular RC buildings on hard, medium, and soft soils, confirming significant reductions in base shear and increases in drift and period due to softer soil.

While extensive research has been conducted on soil amplification effects such as Oz et al. (2020)^53^, Nazar et al. (2025)^54^, Dogan and Erkan (2024)^55^, Rajeev and Tesfamariam (2012)^56^, particularly in non-cohesive soils, cohesive soils and their impact on reinforced concrete (RC) buildings remain underexplored. Existing studies as discussed above focus on buildings of specific heights or symmetrical configurations, neglecting the influence of soil amplification on unsymmetrical structures like L-shaped buildings, which are more susceptible to torsional effects and irregular seismic responses. Additionally, there is limited understanding of how different soil profiles (e.g., clay, sand, gravel, layered soils) and ground motion types (near-field and far-field) affect the seismic performance of multi-story buildings with irregular geometries.

The current study analyzes the soil amplification effect on the seismic vulnerability of L-shaped buildings with various heights (5,10,15, and 20 storeys). Five different soil models (clay, Sand, gravel, Clay-Sand, and Clay-sand-gravel) and 5 different ground motions (3 near-fields and 2 far-fields) are considered to evaluate the soil amplification effect. Pushover and time history analysis have been performed to estimate the seismic response of the building due to the amplified ground motions. The effect of soil amplification on the seismic vulnerability parameters, such as the probability of damage, Damage index, recovery time, and seismic vulnerability index of the building, has been estimated from the fragility analysis. Therefore, this study offers an in-depth examination of how soil amplification of different soil types affects the seismic vulnerability of L-shaped buildings at various building heights. Incorporating soil amplification effects ensures a more accurate evaluation of seismic performance, enabling the design of safer and more resilient structures, especially in regions with soft or heterogeneous soil conditions.

## Building models and design parameters

**Fig. 1:**
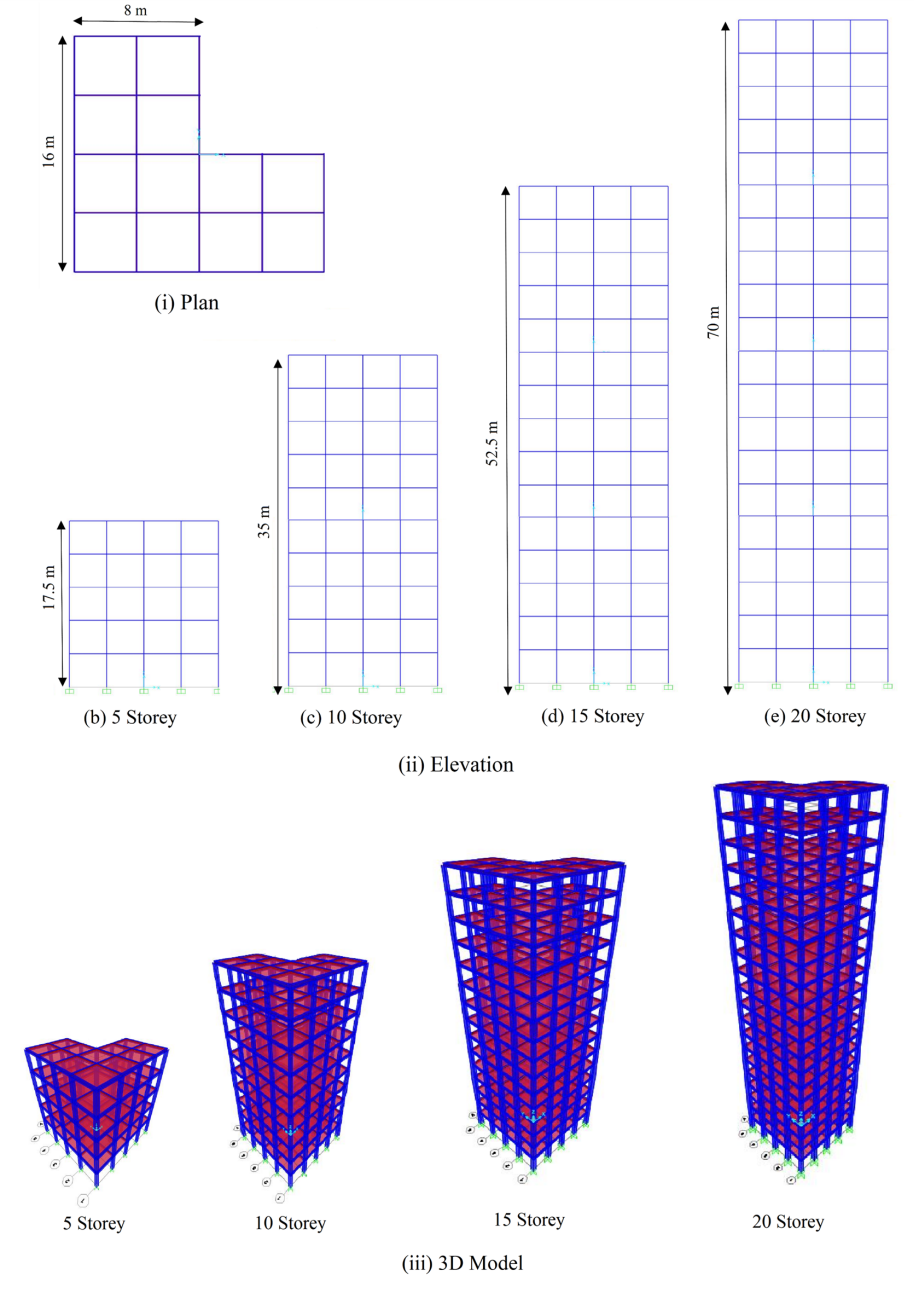
Plan, elevation, and 3D model of the buildings.

The present study considers L-shaped unsymmetrical RC buildings of 5, 10, 15, and 20 storeys with 12.5% plan asymmetry (based on the plan area of the reentrant corner). In this study, the selected reinforced concrete (RC) moment-resisting frame buildings are categorized based on the number of stories into three groups for comparative analysis: low-rise buildings (3–7 stories), medium-rise buildings (8–12 stories), and high-rise buildings (more than 12 stories). This classification aligns with common engineering practice and helps to evaluate the seismic performance trends across different height categories. Accordingly, 5-story (low-rise), 10-story (mid-rise), 15 and 20-story (high-rise) models have been considered, representing each group respectively. The building is modeled in SAP 2000^57^. The plan, elevation, and 3D model of the building are presented in Fig. 1. The building is fixed at the base, and each storey height is 3.5 m. Dead, live, and seismic loading are considered as per IS 875(parts 1 &2):1987^58–60^and 1893 (Part 1): 2016^61^, respectively. The structure is situated in Seismic Zone V as per IS 1893:2016, corresponding to a region of very high seismicity (Zone Factor Z = 0.36). This aligns with Seismic Design Category E or F in FEMA/NEHRP (National Earthquake Hazards Reduction Program)2020^62,63^ and ASCE 7–22^64^ for Risk Category III or IV structures, respectively. The design considers a Response Reduction Factor (R) of 5 and an Importance Factor (I) of 1.5.

**Fig. 2:**
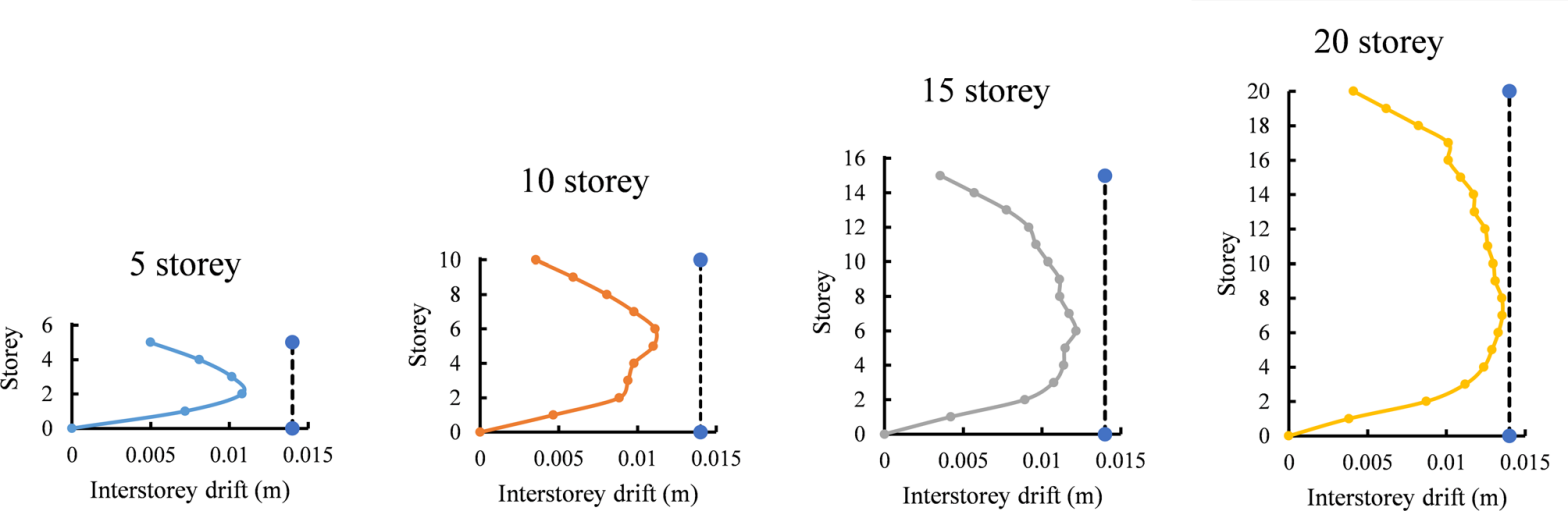
Inter-storey drift check with IS 1893:2016 compliance threshold.

Buildings are designed as per IS 456:2000^59^ (Indian design code) guidelines by considering the concrete grade M 25 (characteristic compressive strength (F_ck_)– 25 N/mm^2^) and steel grade Fe 500 (yield tensile strength (f_y_)- 500 N/mm^2^). Section details of beam and column for 5,10, 15, and 20 storey L shaped buildings are presented in the Table 1. The critical beam and column demand-capacity ratios are less than 1, and the inter storey drift of the building is within the permissible limits as per Indian standards, indicating the structural safety of the building, as demonstrated in Fig. 2. The inter-storey drift profiles shown in Fig. 2 were obtained from linear static analysis (Equivalent Lateral Force Method) as specified in IS 1893 (Part 1): 2016. The analysis was performed for the load combination DL + LL + EL, with a load factor of 1.0 applied to each component, to check the service-level performance of the structures under design seismic conditions. To assess compliance with code-defined drift limits, a vertical dashed line is included at 0.014 m in each subfigure. This value corresponds to the maximum permissible inter-storey drift, calculated as 0.004 times the storey height (0.004 × 3.5 m = 0.014 m), as per Clause 7.11.1 of IS 1893 (Part 1): 2016^61^. The drift profiles for all building configurations remain well within this limit, confirming acceptable lateral performance and structural safety. For the nonlinear analysis, Plastic hinges are assigned to the beam (M3 auto hinge) and column (P-M-M auto hinges) at each end of the member as per ASCE 41^65^. The strong column-weak beam principle has been adopted in this study for all the buildings. This design philosophy ensures that columns are significantly stronger and stiffer than the beams they support, promoting a specific failure hierarchy during seismic events. By designing beams to be weaker, they act as the primary energy dissipation mechanism, undergoing plastic deformation (yielding) before the columns. This controlled yielding in beams absorbs and dissipates seismic energy, preventing brittle failure in columns, which could lead to partial or total collapse of the structure.

**Table 1:** Beam and column properties of the analyzed buildings.

S. No.	Building height	Beam details		Column details		
		Section size	Reinforcement (top & bottom)	Storey	Section size	Reinforcement
1	5 storey	230 × 360	#3–16 mm	1–5 Storey	350 × 350	#10–16 mm
2	10 storey	230 × 370	#3–16 mm	1–5 Storey	470 × 470	#14–16 mm
				6–10 Storey	350 × 350	#10–16 mm
3	15 storey	230 × 380	#3–16 mm	1–5 Storey	570 × 570	#14–20 mm
				6–10 Storey	470 × 470	#14–16 mm
				11–15 Storey	330 × 330	#10–16 mm
4	20 storey	230 × 390	#3–16 mm	1–5 Storey	670 × 670	#20–20 mm
				6–10 Storey	570 × 570	#14–20 mm
				11–15 Storey	470 × 470	#14–16 mm
				16–20 Storey	330 × 330	#10–16 mm

**Fig. 3:**
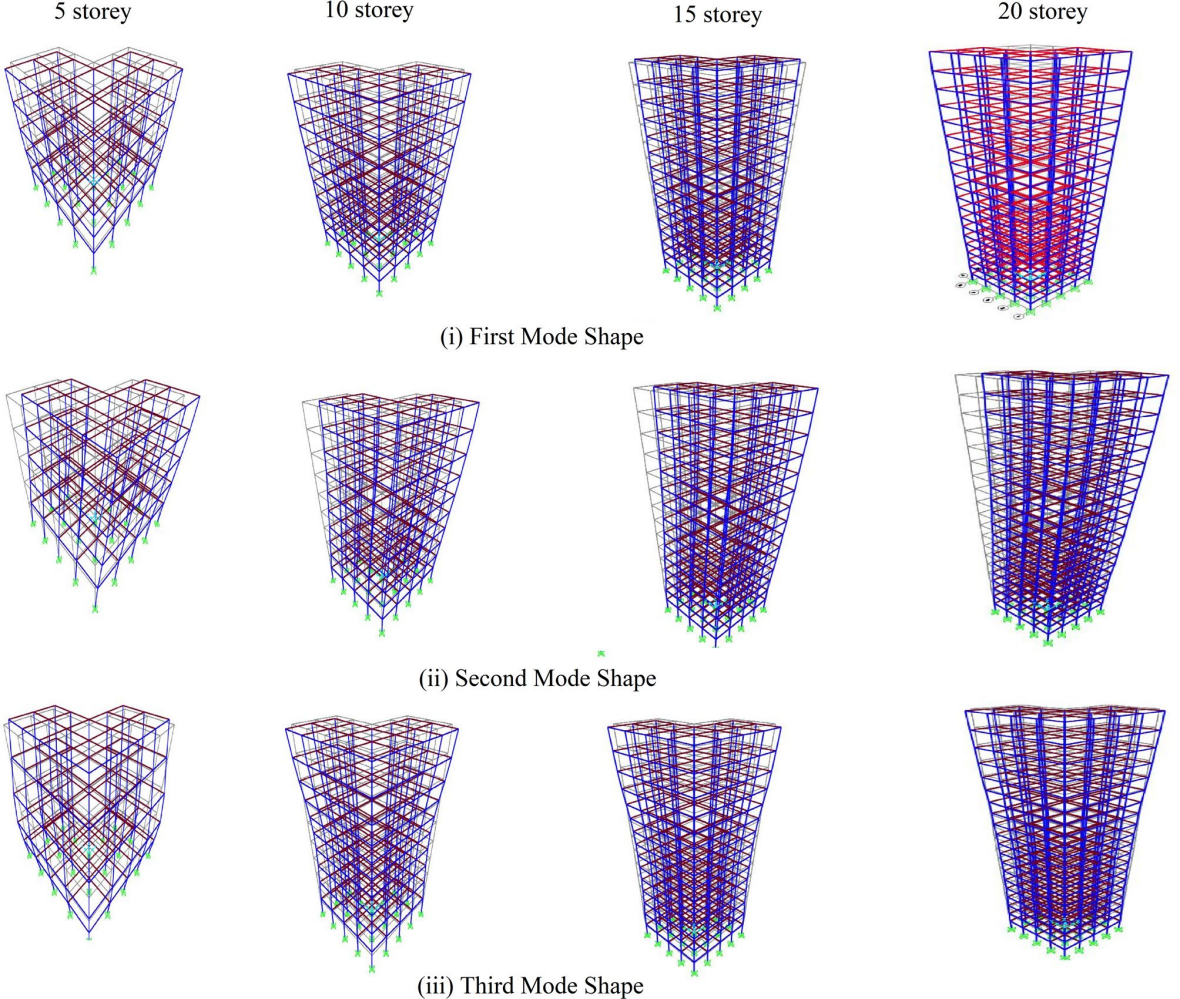
Mode shapes for the first three modes of the 5-, 10-, 15-, and 20-storey L-shaped buildings.

Table 2; Fig. 3 presents the fundamental modal properties—time periods and mass participation ratios—for the first three modes of the analyzed 5-, 10-, 15-, and 20-storey L-shaped buildings. The fundamental time periods increase with building height, ranging from 0.97 s for the 5-storey model to approximately 3.10 s for the 20-storey model, which is consistent with expected dynamic behavior. Notably, the first and second modes exhibit very similar time periods and comparable mass participation ratios across all cases. This indicates coupled lateral–torsional behavior, which is characteristic of buildings with plan irregularities, such as the L-shaped configuration used in this study. The cumulative mass participation in the first three modes exceeds 75% in all models, confirming that the dominant dynamic response is effectively captured.

**Table 2:** Modal time periods (t) and mass participation ratios (M_k_)of the first three modes for 5-, 10-, 15-, and 20-storey L-shaped buildings.

No. of modes	5 storeys		10 storey		15 storey		20 storey	
	t (s)	M_k_	t (s)	M_k_	t (s)	M_k_	t (s)	M_k_
1st mode	0.98	41.5%	1.71	37.9%	2.41	36.9%	3.10	36.1%
2nd mode	0.97	41.3%	1.70	37.3%	2.40	36.3%	3.08	35.6%
3rd mode	0.31	5.2%	0.60	6.2%	0.86	5.8%	1.12	5.9%
Total (top 3 Modes)		88.1%		81.4%		79.0%		77.6%

## Ground motion properties

A total of five real ground motion records were selected for time history analysis, as summarized in Table 3. The ground motion records used in this study were classified as near-fault (≤ 20 km) or far-fault (> 20 km) based on the epicentral distance, following standard practices in seismic analysis (FEMA P695^66^, Chopra 2012^11^. This distinction helps capture the varying effects of pulse-like near-fault motions and smoother far-field shaking. The selection criteria included earthquake magnitudes between 6.0 and 7.2, site conditions matching stiff to medium soils (Type II in IS 1893:2016 / Site Class C–D in NEHRP), and records with at least 1,000 data points. Additionally, the chosen ground motions had predominant periods ranging from 0.18 to 0.64 s, which align with the natural periods of the studied buildings. Only horizontal components were used to ensure consistency in input excitation. This approach ensured realistic and site-compatible ground motions for structural analysis. These ground motions were collected from various seismological stations such as Cosmos and Peer database. Two far fields (GM 1 and GM 2) generally display lower-frequency components and extended durations, and three near-field ground motions (GM 3, GM 4, and GM 5) show high-frequency content and intense velocity pulses. The far-field Indian motions offer regional significance, reflecting local seismic behavior, whereas the near-field motions consider directivity effects and intense energy pulses due to the nearby fault ruptures. Integrating both near-field and far-field ground motions allows for the assessment of soil amplification in various seismic conditions. Thus, these far-field and near-field ground motions guarantee differences in soil response, confirm result consistency, and adhere to seismic code requirements, thus making the findings robust and applicable to various potential earthquake situations.

The selected ground motion records, with peak ground acceleration (PGA) values ranging from 0.06 g to 0.72 g, are consistent with the soil conditions at the building site, which are categorized as medium to stiff soils based on available geotechnical information. These correspond to Type II (medium soil) in IS 1893:2016 and Site Class C–D under NEHRP classification. According to IS 1893:2016^61^, Type II soils (Vs₃₀ between 360 and 760 m/s) typically experience PGAs in the range of 0.2 g to 0.6 g, while softer soils (Type III) may exhibit higher amplification, reaching up to 0.72 g or more. Similarly, NEHRP Site Classes C and D encompass PGA values between 0.2 g and 0.72 g, reflecting moderate to high amplification potential. To ensure the selected records realistically represent seismic demands compatible with the study area’s soil profile, a wide PGA range was included—covering both low-intensity (e.g., GM1 with 0.06 g) and strong shaking scenarios (e.g., GM5 with 0.72 g). This approach captures the possible variability in seismic input and enables a robust assessment of building performance across different intensity levels.

**Table 3:** Characteristics of selected ground motion time histories.

SN.	Ground motion	Station	Earthquake	Magnitude	Distance (km)	PGA	Site class	Predominate period	No. of data points	Type of GM
1	GM 1	Silchar	NE India Earthquake	7.2	247	0.06	C–D	0.38	2338	Far-field
2	GM 2	Silchar	NE India Earthquake	6	89	0.16	C–D	0.64	1346	Far-field
3	GM 3	Coyote Lake Dam Southwest Abutment	Morgan Hill	6.19	12	0.72	C	0.28	5999	Near-field
4	GM 4	Gilroy Array #2	Loma Prieta	6.9	22	0.32	C	0.3	7998	Near-field
5	GM 5	Parkfield-	Cholame 1E	6	14	0.36	C–D	0.18	4263	Near-field

## Soil profile

**Fig. 4:**
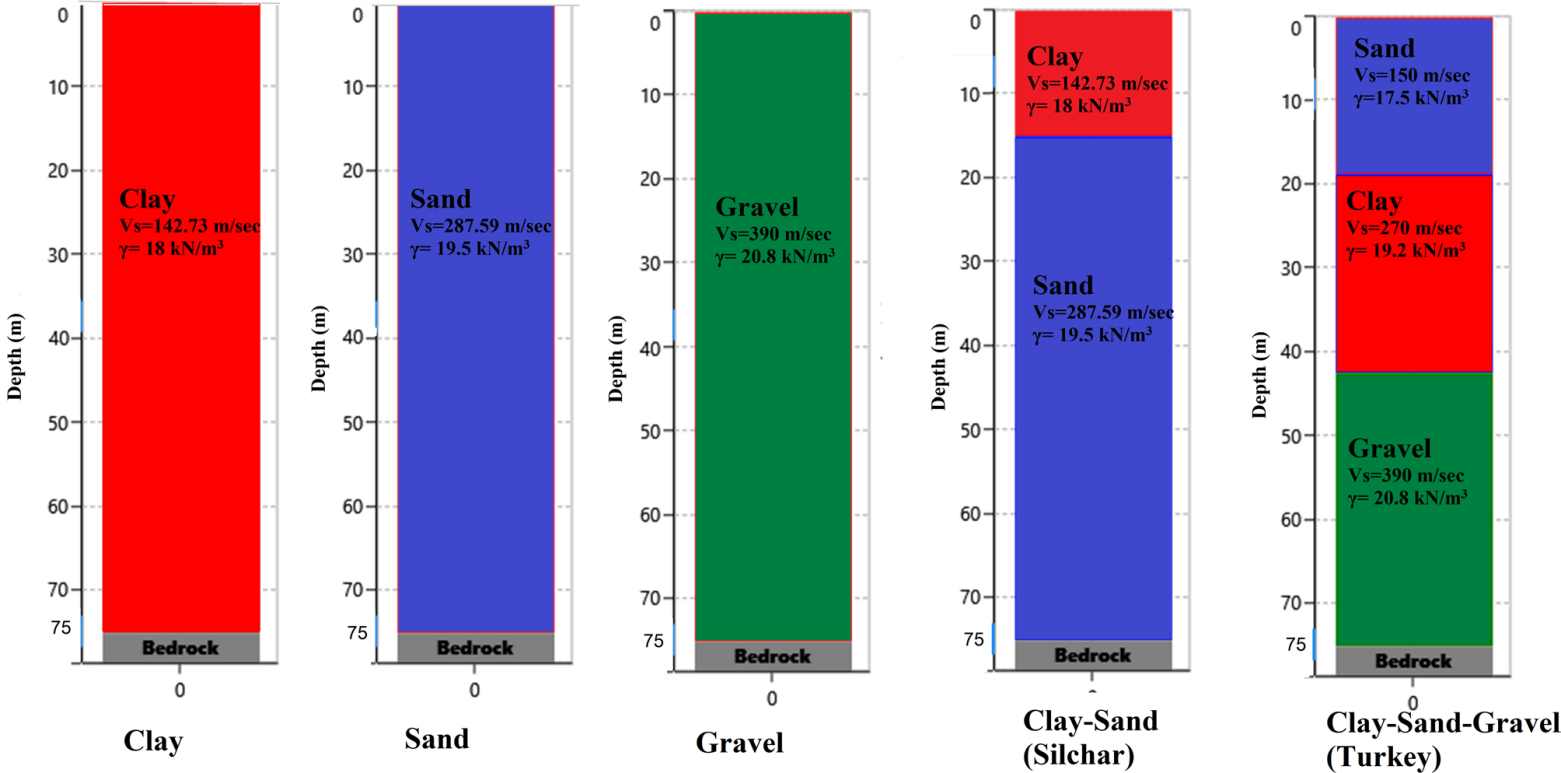
Soil model representing different soil profiles prepared in DeepSoil software.

Five different soil models were analyzed to investigate the effects of soil amplification, as illustrated in Fig. 4. The first three soil profiles represent homogeneous conditions, consisting of pure clay, pure sand, and pure gravel, respectively. The last two profiles are heterogeneous, combining the abovementioned soils: Clay-Sand (Silchar) and Clay-Sand-Gravel (Turkey). The properties of this soil were adopted from previous studies’ borehole data (Özgirgin,1997^67^; Sirgin et al., 2017^68^; Dey & Saha,2023^69^; Day & Paul,2008^70^. In this study, the total soil profile thickness was kept constant at 75 m for all models to allow a consistent comparison of the effects of different soil types (clay, sand, gravel) and regional profiles (Silchar and Turkey). The 75-meter depth for the Silchar site was based on previous geotechnical studies in the region (Dey & Saha,2023^69^; Day & Paul,2008^70^, while the same depth was used for other soil types to maintain uniformity in the analysis. It is noted that while soil thickness does influence amplification, the focus here was on isolating the effect of soil composition under a consistent profile depth. The DEEPSOIL software was used to model the soil profiles. DEEPSOIL is a one-dimensional software for site response analysis that can conduct both nonlinear and equivalent linear assessments. Soil has been modeled in DEEPSOIL by considering various soil properties, including unit weight (γ), shear wave velocity (Vs), thickness of layers, and minimum damping ratio (D_min_) as shown in Fig. 4. Shear wave velocity of various soils ranges from 150 to 390 m/sec, and for bedrock level, it is 760 m/sec. Unit weight ranging from 17.5 to 20.8 kN/m3 minimum damping value (D_min_) ranging from 2 to 5% for various soils. These factors are crucial for effectively modeling the dynamic responses of soils subjected to seismic forces. The research tries to find the impact of varying soil compositions on the amplification of ground vibrations by analyzing these soil models.

## Soil amplification

**Fig. 5:**
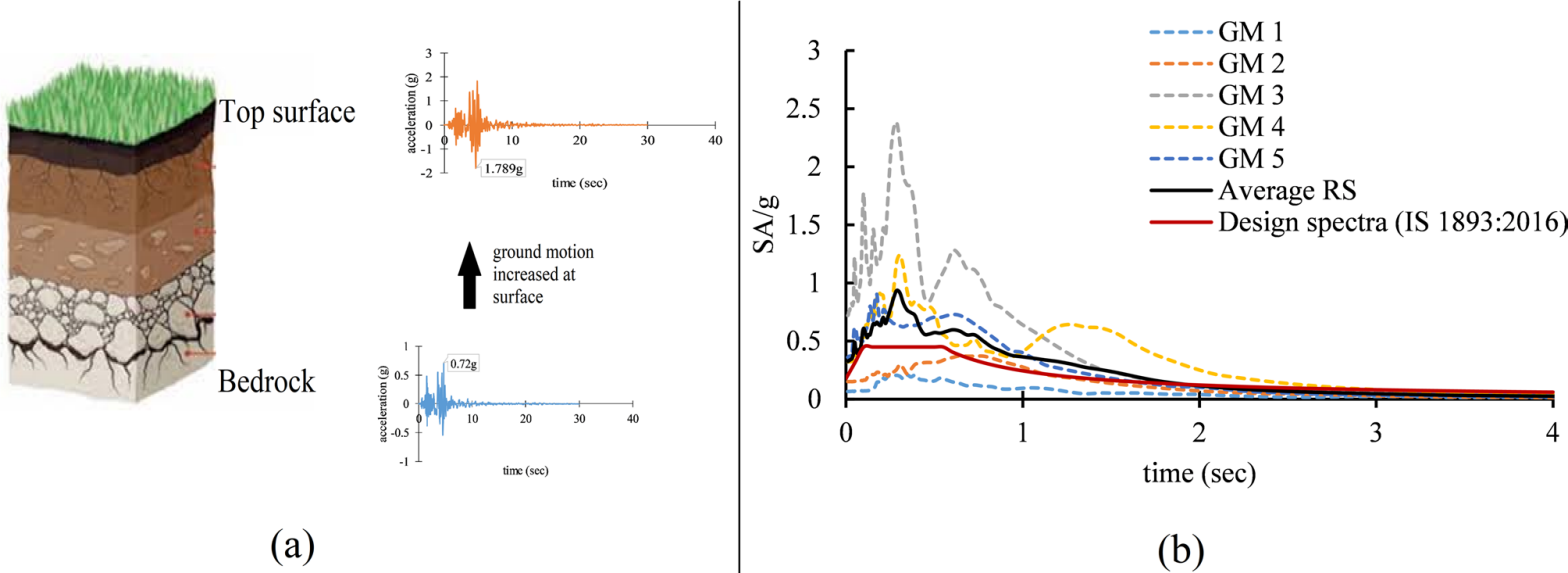
(**a**) Visualization of the soil amplification phenomenon and (**b**).

Soil amplification is the process by which the amplitude of seismic waves increases as they pass through near-surface soil layers, with the extent of amplification depending on the stiffness and layering of the soil, and it is typically more pronounced in soft soils but can also occur in stiffer or rock-like soils, as shown in Fig. 5(a). It can cause significant damage to structures. The soil amplification depends upon various geological and physical factors factor, such as the soil layer’s structure, density, thickness, damping, and wave velocity (Anbazhagan et al., 2021^71^. The ratio of surface wave velocity between the surface soil and base layer is most important in the soil amplification. The presence of structure on the soil also affects the soil amplification. (Lee et al., 2021^72^; Gürtürk and Selçuk,2021^73^. Fine-grained soils and higher plasticity amplify more, while gravelly soils and higher Vs values exhibit lower amplification (Sotiriadis et al. 2017)^38^. The intensity and frequency content of seismic waves further influences amplification, with hills and deeper layers generally resulting in increased peak ground acceleration (PGA) (Horri et al., 2019^74^; Karafagka et al. 2021^75^; Ortiz et al., 2024^76^; Shreyasvi & Venkataramana, 2021^77^. Mainly, analytical models are used to estimate the soil amplification. Field tests that measure and evaluate how sites respond dynamically to artificial excitation, natural forces, and earthquakes provide valuable information for understanding these effects. However, the most reliable assessments of soil amplification are generally derived from examining the recorded ground motion of a site during significant earthquakes since these real-world observations capture the complex interactions between soil properties and seismic waves.

**Fig. 6:**
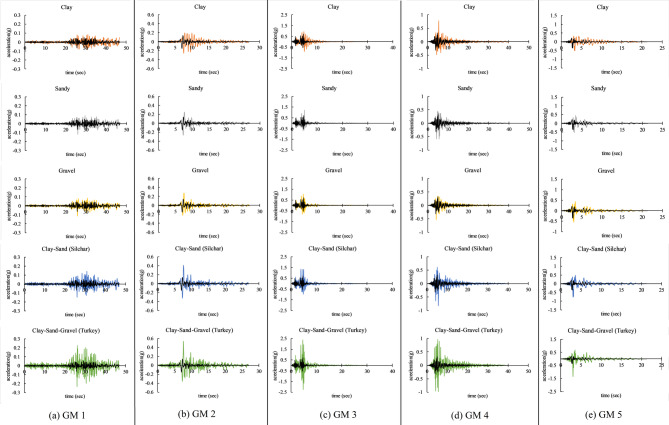
Effects of soil amplification on ground motions across soil profiles.

**Fig. 7:**
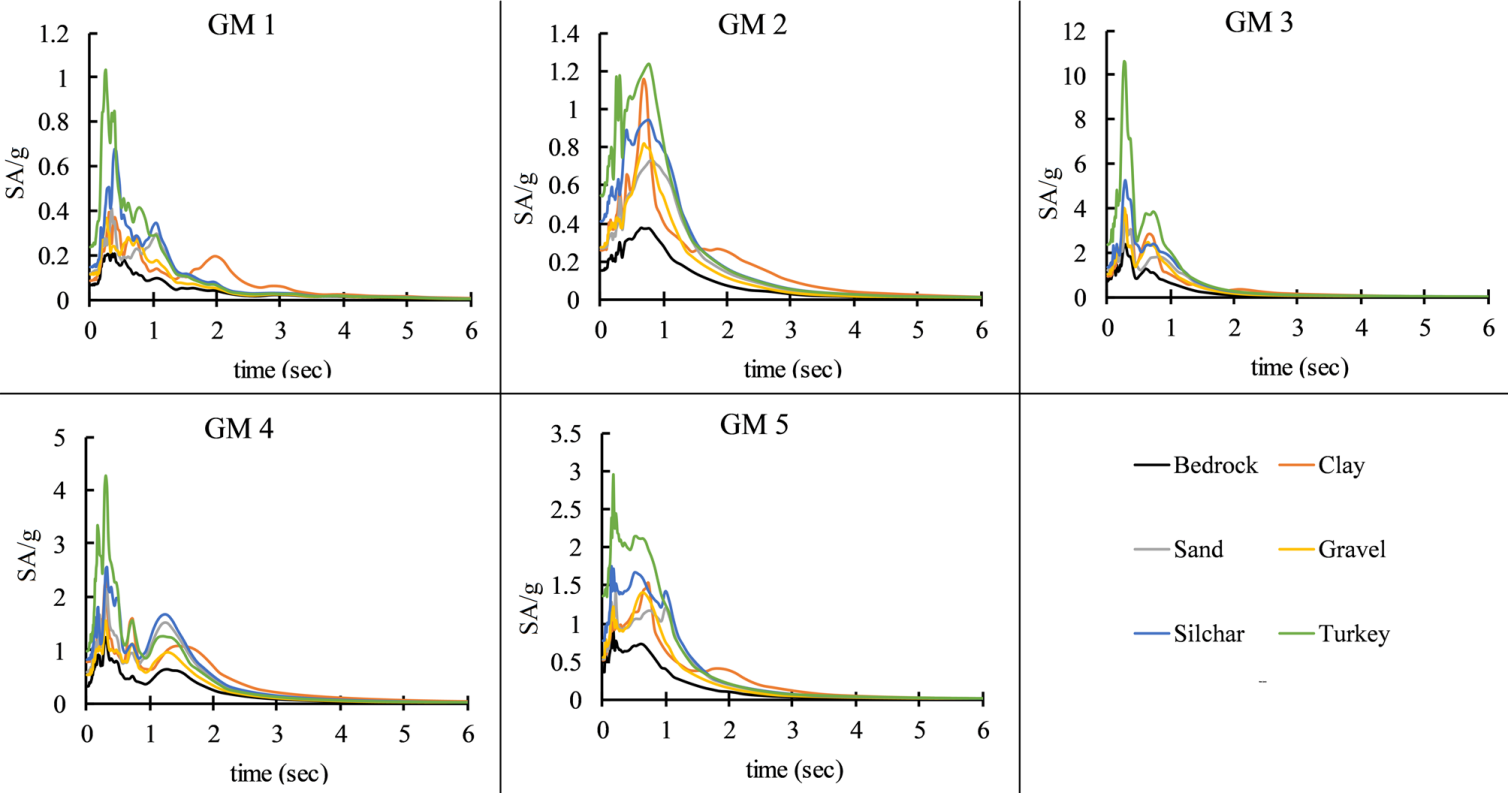
Influence of soil amplification on response spectra for various soil conditions.

Here, after modeling the various soil profiles in DEEP SOIL software, all the 5-time histories were applied at the bedrock level. In this study, the ground motions were used in their recorded form and were not modified to match the design response spectrum as per IS 1893:2016^61^. This decision was made to retain the inherent variability and characteristics of real earthquake records, allowing for a more realistic evaluation of structural response under actual seismic inputs, rather than artificially adjusted ones. The spectral acceleration curves of all selected ground motion records along with their average have been compiled and presented in a single graph, as shown in Fig. 5(b). This allows for a direct comparison of the ground motion characteristics and supports a clearer interpretation of the variability relative to the design spectrum. Figure 6 illustrates the soil amplification of Far-field (GM1 and GM2) and near-field ground motion due to clay, sand, Gravel, Clay-Sand (Silchar), and Clay-Sand-Gravel (Turkey) soil profiles. The original ground motion represents ground motion at the bedrock level, which is denoted by the black color in the figure. Various color lines show the amplified ground motion due to various soil profiles. The results show that the time histories and response spectrum of all far field and Near field ground motion are significantly increased at the surface level due to the soil amplification effect as compared to the bedrock level, as presented in Figs. 6 and 7. The maximum amplification is observed in mixed-type soils, such as in Turkey, with a peak ground acceleration (PGA) ratio of approximately 3.3 and a peak spectral acceleration (PSA) ratio of around 3.6 relative to the original input motion. In contrast, the minimum amplification is seen in pure gravel soils, with a PGA ratio of about 1.3 and a PSA ratio of approximately 1.7, as detailed in Table 4. Table 4 represents the soil amplification effect on Peak ground acceleration value (PGA) and Peak spectral acceleration value (PSA). Soil amplification can increase PGA values to 0.171–0.393 g (far-field) and 0.66–1.649 g (near-field), while PSA values may be increased to 0.82–0.85 g (far-field) and 2.05–6.23 g (near-field). Here, it has been observed that Clay soil profile can increase the PGA value by up to 42–141%, Sandy soil can increase by 46–83%, Gravel soil can increase the 31–69%, Silchar soil profile can increase up to 87–168%, Turkey soil profile can increase up to 204–275%. Similarly, the PSA value can increase up to 57%-126% for clay, 41–92% for sand, 25–78% for gravel, 94–223% for Silchar, and 227–390% for Turkey soil. The results show that clay and layered soils (e.g., Silchar, Turkey) exhibit higher PGA and PSA amplification, especially in the 0–4 s period range, making them more vulnerable. In contrast, sand and gravel profiles show minimal amplification across all periods.

**Table 4:** Peak ground acceleration (PGA) and peak spectral acceleration (PSA) at bedrock and surface levels.

S. No.	Soil	PGA (g)					PSA (g)				
		GM1	GM2	GM3	GM4	GM5	GM1	GM2	GM3	GM4	GM5
1.	Original	0.067	0.153	0.720	0.323	0.362	0.21	0.38	2.38	1.24	0.90
2.	Clay	0.115	0.274	1.043	0.780	0.517	0.39	1.16	3.75	2.29	1.53
3.	Sand	0.121	0.269	1.223	0.592	0.529	0.41	0.73	3.37	2.14	1.42
4.	Gravel	0.087	0.259	0.948	0.539	0.555	0.37	0.82	4.01	1.55	1.41
5.	Clay–Sand (Silchar)	0.148	0.410	1.352	0.826	0.773	0.68	0.94	5.26	2.56	1.75
6.	Clay–Sand–Gravel (Turkey)	0.238	0.546	2.369	0.983	1.358	1.03	1.23	8.61	4.27	2.95

## Methodology

**Fig. 8:**
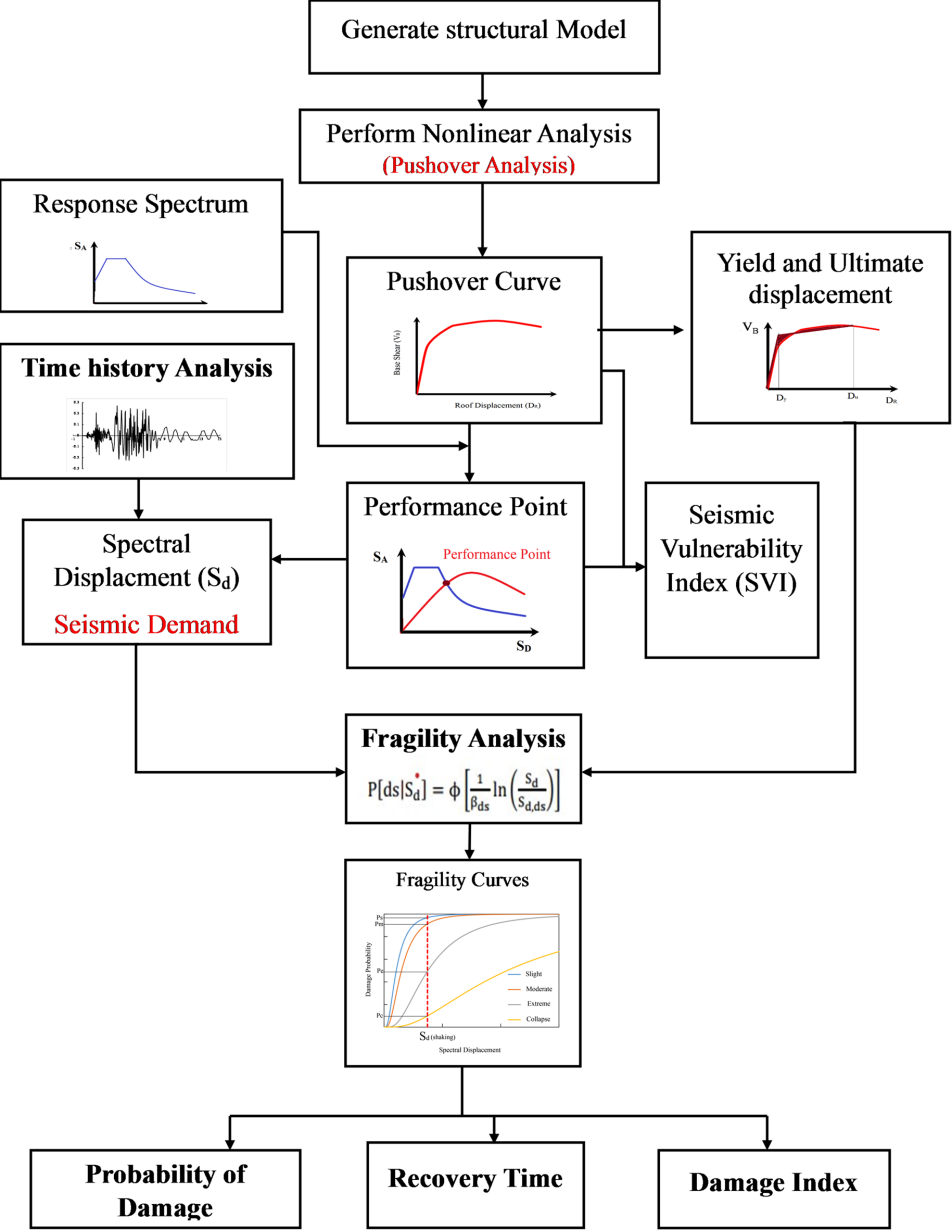
Methodology for assessing seismic vulnerability of buildings.

The methodology of the seismic vulnerability assessment of the building includes pushover analysis, time history analysis, and fragility analysis of the building. The flowchart of the methodology is shown in Fig. 8. Pushover analysis was conducted in both x and y directions to estimate global capacity points (yield and ultimate displacement/base shear) for fragility curve development. Despite the building’s torsional irregularity, pushover remains suitable for global capacity estimation when interpreted cautiously (FEMA-356, 2000^78^; Chopra & Goel, 2002^79^. Time history analysis was separately performed to capture seismic demand under various ground motions, including torsional effects. Fragility analysis provides a seismic vulnerability assessment of the building, which includes the probability of damage, damage index, recovery time, and seismic vulnerability index.

### Fragility analysis

The Fragility analysis involves the generation of fragility curves of the various damage states: slight, moderate, severe, and collapse). The fragility curves can be used to estimate the structural seismic vulnerability. Fragility curves quantify the probability of different damage states occurring at specified intensity levels (Kumar et al., 2022 & 2024^80,81^; Kumar & Ghosh, 2024 & 2025^82,83^; Kassem et al., 2020^84^. Fragility curves are the diagram that shows the relationship between the cumulative probability of damage to the particular intensity measure, such as spectral displacement, spectral acceleration, and spectral velocity. In past studies, fragility curves have been developed for various building types, including reinforced concrete structures, highlighting the need to retrofit older buildings to meet current seismic codes (Souki et al., 2024^85^; Gubana & Mazelli, 2023^86^. The present study uses the analytical method for the generation of fragility curves, as shown in Fig. 8.

As per the Hazus manual, the slight Damage is characterized by flexural or shear-type hairline cracks in some beams and columns near or within joints, indicating minor structural distress with minimal repair needs. Moderate Damage involves most beams and columns exhibiting hairline cracks; in ductile frames, some elements reach yield capacity, evidenced by larger flexural cracks and some concrete spalling, while nonductile frames may show larger shear cracks and spalling, requiring more substantial repairs. Extreme Damage occurs when some frame elements reach their ultimate capacity, with ductile frames showing large flexural cracks, spalled concrete, and buckled main reinforcement, and nonductile frames experiencing shear failures, bond failures at reinforcement splices, broken ties, or buckled column reinforcement, potentially leading to partial collapse. Complete Structural Damage is defined by the structure having collapsed or being in imminent danger of collapse due to brittle failure of nonductile frame elements or loss of overall frame stability, rendering the building uninhabitable.

Yield and Ultimate spectral displacement of the building and spectral displacement value are primary inputs to estimate the probability of damage. The cumulative probability of damage in various states can be estimated from the Hazus manual^87^.

1 $$\:\text{C}\left[\text{d}\text{s}|{\text{S}}_{\text{d}}\right]={\upvarphi\:}\left[\frac{1}{{{\upbeta\:}}_{\text{d}\text{s}}}\text{l}\text{n}\left(\frac{{\text{S}}_{\text{d}}}{{\text{S}}_{\text{d},\text{d}\text{s}}}\right)\right]$$

Where C_di_ represents the Cumulative probability of damage in i^th^ damage state. S_d_ is the spectral displacement, S_dds_ is the median value of spectral displacement, and β_ds_ is the standard deviation, and φ is the lognormal cumulative distribution function. As proposed by Barbat et al. (2008)^88^ the median spectral displacement (S_dds)_ value corresponding to different damage states can be estimated based on the yield displacement (S_dy_) and ultimate displacement (S_du_) obtained from the bilinear idealization of the building’s pushover curve. Specifically, the median spectral displacement is taken as 0.7 × S_dy_ ​ for the **Slight** damage state, S_dy_ for **Moderate** damage S_dy_+0.25 × (S_du_- S_dy)_ for **Extensive** damage, and equal to S_du_ for the **Collapse** damage state.

In the context of HAZUS fragility curves, the β_ds_ value represents the total uncertainty in the spectral displacement at which a specific damage state (e.g., slight, moderate, extensive, complete) is reached. These values quantify variability from three sources: capacity (e.g., differences in material properties or construction quality), demand (e.g., variations in ground motion or site effects), and modeling (e.g., simplifications in analytical models like single-degree-of-freedom systems). These values can be adopted directly from the HAZUS technical manual or customized through statistical methods if site-specific data is available. In the current study, these values are taken from the Hazus manual and which are depends upon the various factor such as seismic zone, type and height of the building (Hazus MH 2.1. 2003)^87^. In this study, the RC frame buildings are classified as Type C1 according to the HAZUS methodology, with mid-rise (C1M, 5 stories) and high-rise (C1H, 10–20 stories) configurations. As per the Indian seismic code (IS 1893:2016), the study area falls under Seismic Zone V, which represents the highest level of seismic hazard. Therefore, in alignment with HAZUS classifications, Zone V is assumed to be equivalent to a high-code seismic design level, and accordingly, high-code building provisions have been adopted in this study. As per (Table 5.12) of the HAZUS Technical Manual, the β_ds_ values for high-code buildings in high seismicity regions are 0.68, 0.67, 0.68, and 0.81 for C1M, and 0.66, 0.64, 0.67, and 0.78 g for C1H, corresponding to Slight, Moderate, Extensive, and Complete damage states, respectively. The fragility curves can be developed using the median spectral displacement values (S_dds)_ and the corresponding logarithmic standard deviations (β_ds_) for each damage state. These curves are then used to estimate the cumulative damage of the building by evaluating the probability of exceeding each damage state at various spectral displacement values. The parameter extracted from the Time History (TH) analysis is the maximum roof displacement corresponding to both the original and the amplified ground motion records. To obtain the spectral displacement, this maximum roof displacement is converted using results from modal analysis, specifically by relating it to the fundamental mode shape of the structure. This approach allows for a consistent comparison of seismic demand in terms of spectral displacement, even when derived from time history simulations.

#### Damage index and recovery time

The Damage Index is a quantitative representation of overall damage to building that tells us how much damage a building might experience during an earthquake. It is calculated as a weighted average of different damage states, based on their probabilities. The Damage Index values vary from 0 to 4, indicating building damage levels during an earthquake: 0 to 0.5 for no damage, 0.5 to 1.5 for slight damage, 1.5 to 2.5 for moderate damage, 2.5 to 3.5 for severe damage, and 3.5 to 4.0 for collapse damage. Each range shows the expected structural damage, from no damage to complete collapse (Barbet et al.,2008)^88^. Similarly, recovery time shows the time required to recover a building after a particular earthquake. Damage Index (DI) and Recovery Time of the building can be estimated by using the following equation:

2 $$\:{\text{D}\text{I}}_{\text{i}}=\sum\:_{\text{i}=1}^{4}{\text{D}}_{\text{i}}\text{*}{\text{P}}_{\text{d}\text{i}}$$

3 $$\:{\text{R}\text{T}}_{\text{i}}=\sum\:_{\text{i}=1}^{4}{\text{T}}_{\text{i}}\text{*}{\text{P}}_{\text{d}\text{i}}$$

Here D_i_, T_i_ is weighted given to each damage state considered from the Barbet et al. (2008) and Hazus Manual. P(d_si_) is discrete probability of damage to the building as presented in the Table 5.

**Table 5:** Weighting factors for damage States and performance level.

Damage state	Damage Index (Di)	Recovery time (Ti)	Seismic Vulnerability Index	
	Barbet et al. (2008)^49^	(Hazus manual: Table 11.8)	Performance level (i)	Weighted Factor (x_i_)
Slight	1	10	B-IO	0.125
Moderate	2	120	IO-LS	0.425
Extreme	3	480	LS-CP	0.775
Collapse	4	960	CP<	1.0

#### Seismic vulnerability index

**Fig. 9:**
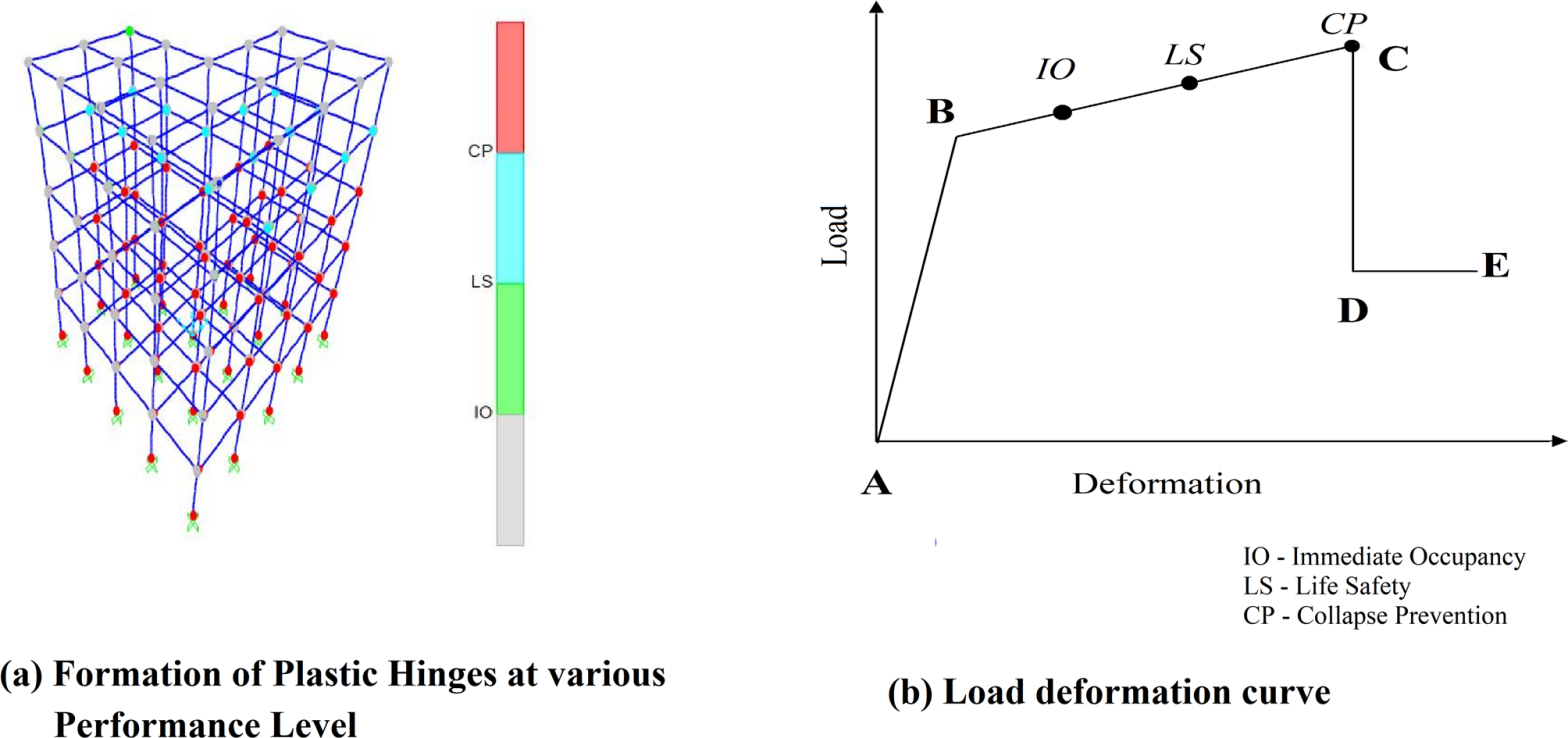
(**a**) Graphical representation of plastic hinge formation and (**b**) load deformation curve showing various performance level.

The Seismic Vulnerability Index is a parameter used to assess the performance of a building after an earthquake, considering the importance of frame elements and the formation of plastic hinges in the beam and column at different performance levels. Pushover analysis can be used to find the number of plastic hinges forming in the structure due to the earthquake. SVI is determined by counting the number of plastic hinge formations in building elements at various performance levels (B-IO, IO-LS, LS-CP, CP< ) and then multiplying them by weighted factors. The load-deformation curve is a vital tool for evaluating structural performance under increasing lateral loads. It is divided into several key phases that represent different performance levels. Initially, the elastic range (A–B) corresponds to reversible deformations before yielding occurs. Beyond this, the plastic region (B–C) is segmented into distinct performance stages: Immediate Occupancy (B–IO), where the structure experiences minimal damage and remains functional; Life Safety (IO–LS), indicating moderate damage with ensured occupant safety; and Collapse Prevention (LS–CP), where the structure sustains severe damage but avoids total failure. The post-collapse region (CP< ) includes stages such as strength degradation (C–D) and residual strength (D–E), reflecting the reduction in load capacity and remaining structural integrity under diminished demand as shown in the Fig. 9. Figure 9a shows the graphical representation of hinge formation in various performance level and Fig. 9b shows the description of various performance on load deformation curve.

The following equation can be used to estimate the SVI of the building (Kassem et al., 2019;2023^89,90^.

4 $$\:{\text{S}\text{V}\text{I}}_{\text{b}\text{u}\text{i}\text{l}\text{d}\text{i}\text{n}\text{g}}=\frac{1.5\sum\:{\text{N}}_{\text{i}}^{\text{c}}{\text{x}}_{\text{i}}+\sum\:{\text{N}}_{\text{i}}^{\text{b}}{\text{x}}_{\text{i}}}{\sum\:{\text{N}}_{\text{i}}^{\text{c}}+\sum\:{\text{N}}_{\text{i}}^{\text{b}}}$$

Here N_i_^c^ and N_i_^b^ and are the number of plastic hinges formed in column and beam and i is the performance level from the 1–4. Weighted factor corresponding to the various performance level are presented in the Table 5. The Seismic Vulnerability Index (SVI) ranges from 0.1 to 1.0, indicating building vulnerability levels: 0.1–0.15 (Green 1, negligible damage), 0.15–0.40 (Green 2, light damage), 0.40–0.55 (Orange 3, moderate), 0.55–0.70 (Orange 4, severe damage), and 0.70–1.00 (Red 5, total collapse). (Belheouane and Bensaibi, 2013^91^.

## Results and discussions

### Response spectrum (SA value)

**Fig. 10:**
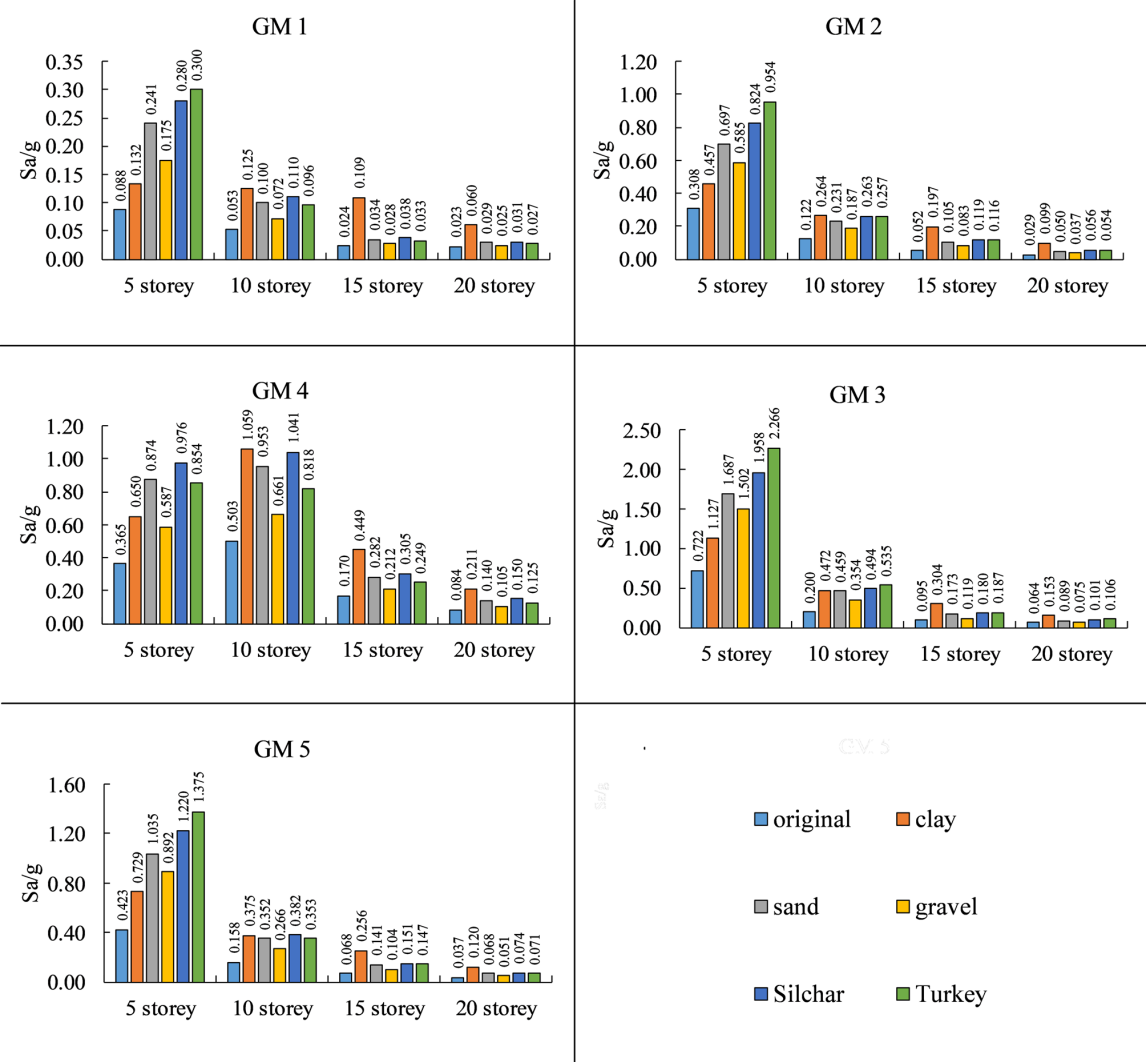
Spectral acceleration (SA) values corresponding to the fundamental period of the building estimated from the Response spectrum of various ground motion (original and amplified).

The spectral acceleration values corresponding to the 1st mode period (fundamental period) from the response spectrum of original and amplified ground motion for different heights of the building, as shown in Fig. 10. The near-field ground motion shows a significant increase (up to 3 times) in spectral acceleration value compared to the far-field ground motion. It has been observed that SA value has increased up to 0.045–0.646 g for 5 storey, 0.019–0.142 g for 10 storey, 0.004–0.145 g for 15 storey, and 0.002–0.07 g for 20 storey building in far-field ground motion and to 0.222–1.544 g for 5 storey, 0.108–0.556 g for 10 storey, 0.024–0.279 g for 15 storey, and 0.011–0.129 g for 20 storey building in near field ground motion. Similarly, soil amplification is greatly affected by different soil types and their combination.

The result shows that the clay soil profile increases the spectral acceleration (SA) value by 78–350%, Sandy soil by 84–175%, gravel soil by 37–110%, Silchar soil by up to 100–220%, and Turkey soil by up to 92–245%. This increase in SA value varies with building height. For example, for low-to-rise buildings (5 storey), the maximum increase in SA value is observed for the Turkey soil profile (160–245%) compared to clay (78–138%). However, as the building height increases, pure clay shows the maximum increase in SA value (240–355%) compared to Silchar and Turkey soil profiles (92–127%). Pure gravel shows the minimum increase in SA value, ranging from 76 to 110% for low- to mid-rise buildings and 37–59% for high-rise buildings. Thus, for low- to mid-rise buildings, layered soil is more critical compared to pure clay, sand, and gravel. On the other hand, for high-rise buildings, pure clay is more critical than Silchar and Turkey soil profiles.

### Pushover analysis

In this study, nonlinear static pushover analysis was performed to estimate the global seismic capacity of the building, specifically to identify key performance points such as the yield and ultimate base shear and displacement capacities. These capacity parameters were then used to define damage states for the development of analytical fragility curves. The pushover method provides a computationally efficient approach to evaluate the nonlinear behavior of the structure under lateral loads and is widely used in performance-based seismic assessment frameworks (FEMA-356, 2000^78^; ATC-40, 1996^92^.

Although the studied structure exhibits plan asymmetry and potential torsional behavior, the objective of the pushover analysis was not to capture detailed dynamic or localized responses but to characterize the global capacity curve of the system. As demonstrated by Krawinkler and Seneviratna (1998)^93^ and Chopra and Goel (2002)^79^, pushover analysis remains useful for irregular and unsymmetrical buildings when applied with appropriate interpretation and limitations. To account for the torsional effects to some extent, pushover analyses were performed in both principal orthogonal directions.

Modal analysis confirms that torsional components are present in early modes to address this, nonlinear time history (TH) analysis was carried out to obtain the seismic demand, particularly the maximum roof displacement under a suite of ground motions. The comparison of TH-based demand with pushover-derived capacity thresholds forms the basis of fragility curve generation. This dual approach—using pushover for capacity and TH analysis for demand—has been widely adopted in seismic vulnerability studies (Calvi et al., 2006^94^; Porter et al., 2007^95^ and balances computational efficiency with sufficient accuracy for system-level performance evaluation.

**Fig. 11:**
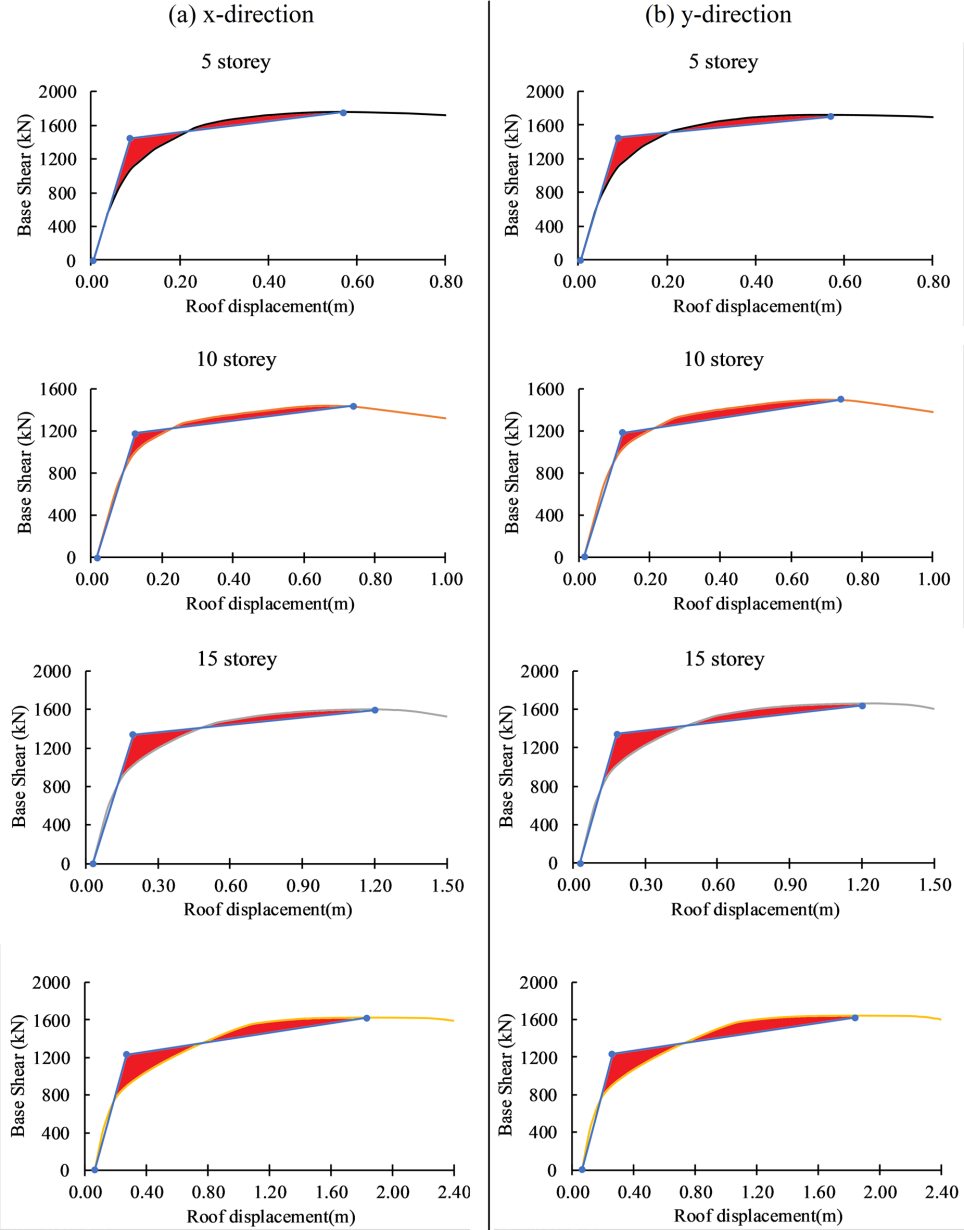
Pushover curves and bilinearization of pushover curve illustrating structural performance of the building in x and y direction.

In pushover analysis, incrementally lateral load (triangular) has been applied throughout the height of the building until the structure reaches its maximum capacity, and output is recorded in terms of base shear and roof displacement (top most point of the building). The curve between the base shear and roof displacement is called the pushover curve or capacity curve. The pushover curve of the 5, 10, 15, and 20-storey buildings in the x and y directions are presented in Fig. 11, which shows the base shear and roof displacement capacity of the building. The y-direction pushover curve shows a higher Ultimate base shear value as compared to the x-direction, although this difference is minimal (20-60kN). Similarly, the ultimate roof displacement capacity of the building in the y direction is higher than in the x direction. The result shows that the ultimate roof displacement of the building increased with building height, which shows that rising buildings are more flexible and lead to increases in drift under the earthquake loading. Yield and ultimate displacement of the building can be calculated from the bilinearization of the pushover curve as per ATC 40 by using the equal area method. Yield and ultimate displacement are the input parameters for the generation of the fragility curve.

### Time history analysis

The seismic response has been estimated through the time history analysis of 5,10,15, and 20-storey L-shaped RC buildings. A total of five-time histories (comprising 2 far-field and 3 near-field records) are applied at the bedrock level. These time histories are then amplified at the surface level due to different soil profiles, including clay, sand, gravel, Silchar, and Turkey soil. As a result, six ground motion cases are considered for the time history analysis: one corresponding to the bedrock level ground motion and five corresponding to the surface level ground motions. The time history analysis is conducted using SAP 2000 software, and the building’s response is evaluated in terms of roof displacement, spectral displacement, ductility demand, and base shear capacity.

A total of five histories (2 far fields and 3 near fields) are applied to the building of different heights at the bedrock level, and their amplified time history at a surface level due to clay, sand, gravel, Silchar, and Turkey soil profile are observed. Thus, a total of six ground motion cases are used for time history analysis: one is bedrock level ground motion, and 5 are the surface level ground motion. The time history analysis has been performed by using the SAP 2000 software, and the response of the building is observed in terms of roof displacement, spectral displacement, ductility demand, and base shear capacity of the building. The parameter extracted from the Time History (TH) analysis is the maximum roof displacement corresponding to both the original and the amplified ground motion records. To obtain the spectral displacement, this maximum roof displacement is converted using results from modal analysis, specifically by relating it to the fundamental mode shape of the structure. This approach allows for a consistent comparison of seismic demand in terms of spectral displacement, even when derived from time history simulations.

#### Roof displacement and spectral displacement

**Fig. 12:**
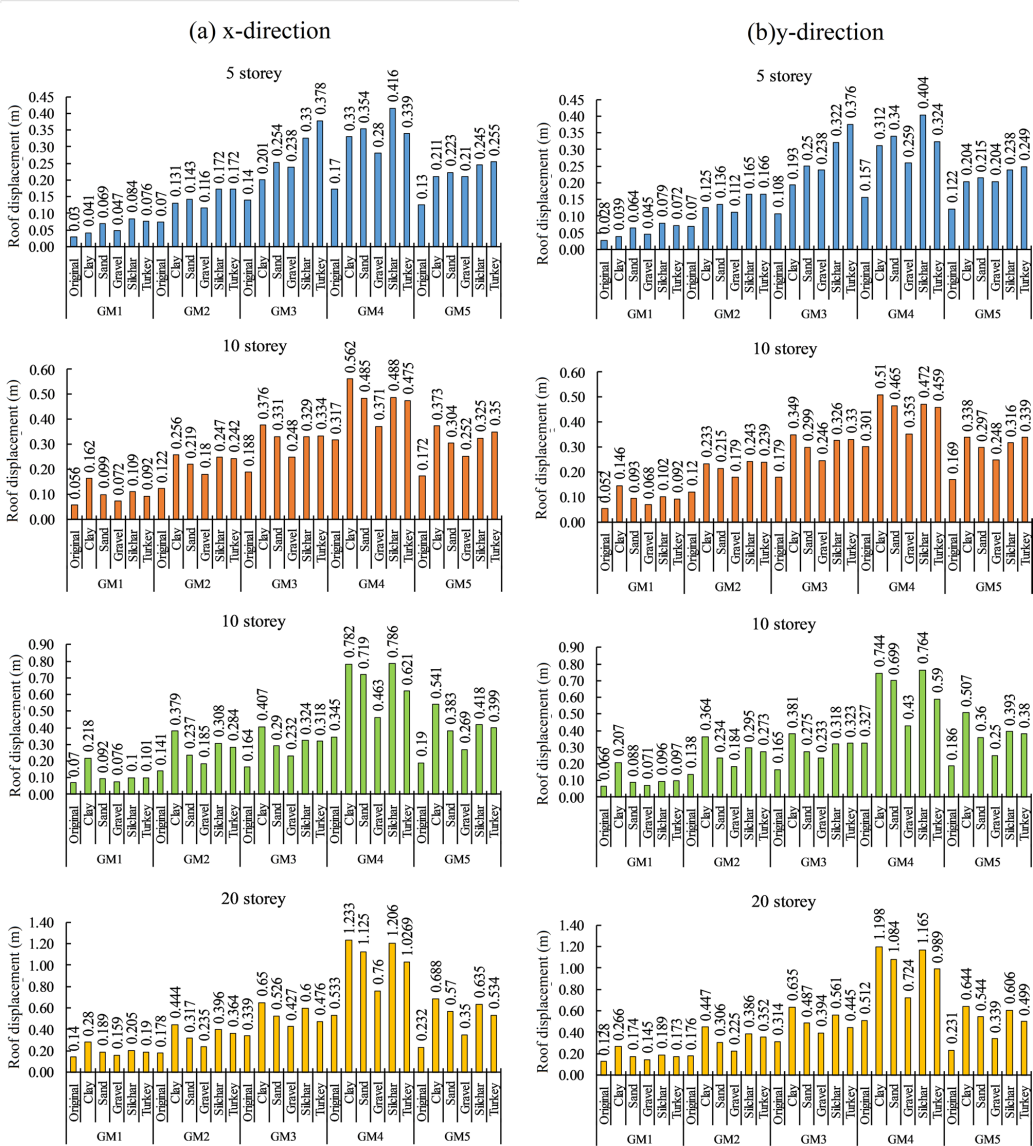
Roof displacement (m) comparison for bedrock and surface-level ground motions in x and y directions.

The maximum roof displacement of 5, 10, 15, and 20-storey buildings due to bedrock level and surface level ground motion for different soil profiles are presented in Fig. 12. The roof displacement of the building significantly increased due to the soil amplification effect. The increment in roof displacement is observed more in near-field ground motion than in far-field ground motion. The result shows that for the far field ground motion, the roof displacement value has increased by up to 12–99 mm in 5 storeys, 16–134 mm in 10 storey, 15–238 mm in 15 storey, and 19–266 mm in 20 storey building and for near field ground motion the roof displacement value has increase by up to 62–244 mm in 5 storey, 54–245 mm in 10 storey, 68–441 mm in 15 storey, and 88–700 mm in 20 storey building. Thus, increments in roof displacement value due to soil amplification are 80–160% higher in near-field ground motion as compared to the far-field ground motion. Similarly, the seismic response of the buildings increased due to soil amplification for all soil profiles. The result shows that roof displacement values are increased by up to 79–211% in clay, 68–146% in sand, 31–71% for gravel, 89–190% for Silchar, and 98–170% for the Turkey soil profile. For low-rise buildings (5 storey), the combination of soil profiles (Silchar and Turkey) resulted in the maximum increase (162–190%) in roof displacement compared to clay (79%) and sand (138%). On the other hand, for mid to high-rise buildings (10 to 20 storeys), clay soil caused the maximum increase (149–211%) compared to the combination of soil profiles (98–122%). Additionally, the Roof displacement of the building significantly changes with building height. It was seen that the roof displacement value has been increased by 62–190% for 5 storeys, 47–189% for 10 storeys, 31–211% for 15 storeys, and 32–197% for 20-storey buildings. The spectral displacement value of the different heights of the building for various cases is estimated from the roof displacement by using a modal analysis of the building, as presented in Table 6. x-direction roof displacement value is higher than the y-direction roof displacement, up to 22.3%. The spectral displacement value of the different heights of the building for various cases is estimated from the roof displacement by using a modal analysis of the building. These spectral displacements are the primary input to estimate the probability of damage to the building as it shows the seismic demand of the building for fragility analysis.

**Table 6:** Spectral displacement (mm) of buildings under bedrock and surface-level ground motions in x and y directions.

Ground motion	Soil profile	x-direction				y-direction			
		5 storey	10 storey	15 storey	20 storey	5 storey	10 storey	15 storey	20 storey
GM1	Original	18	30	29	54	19	29	28	47
	Clay	28	115	148	170	27	107	145	163
	Sand	50	63	45	94	49	62	45	85
	Gravel	33	42	33	69	33	41	32	61
	Silchar	62	71	51	107	61	69	52	98
	Turkey	56	58	52	95	55	61	53	84
GM2	Original	54	82	84	85	54	85	87	87
	Clay	101	194	283	307	100	181	278	315
	Sand	111	163	164	202	109	166	168	197
	Gravel	89	130	120	132	89	135	126	128
	Silchar	135	187	224	268	133	189	220	264
	Turkey	135	183	204	241	134	186	202	236
GM3	Original	108	137	103	220	86	135	110	204
	Clay	158	290	306	477	155	273	292	470
	Sand	200	253	209	375	200	235	203	348
	Gravel	187	188	160	293	190	192	168	271
	Silchar	258	251	237	436	258	255	239	409
	Turkey	299	256	232	334	301	258	244	313
GM4	Original	135	242	255	381	127	236	247	369
	Clay	260	444	602	935	250	406	578	913
	Sand	279	380	552	854	273	369	544	829
	Gravel	220	286	352	566	207	276	332	542
	Silchar	329	382	605	914	324	374	594	889
	Turkey	267	371	476	778	260	364	462	754
GM5	Original	96	124	125	130	97	127	128	133
	Clay	166	287	414	508	164	264	395	477
	Sand	175	232	286	411	173	233	274	395
	Gravel	165	191	191	229	164	193	182	225
	Silchar	193	248	315	464	190	248	302	446
	Turkey	200	268	299	382	199	265	291	358

#### Ductility demand

**Fig. 13:**
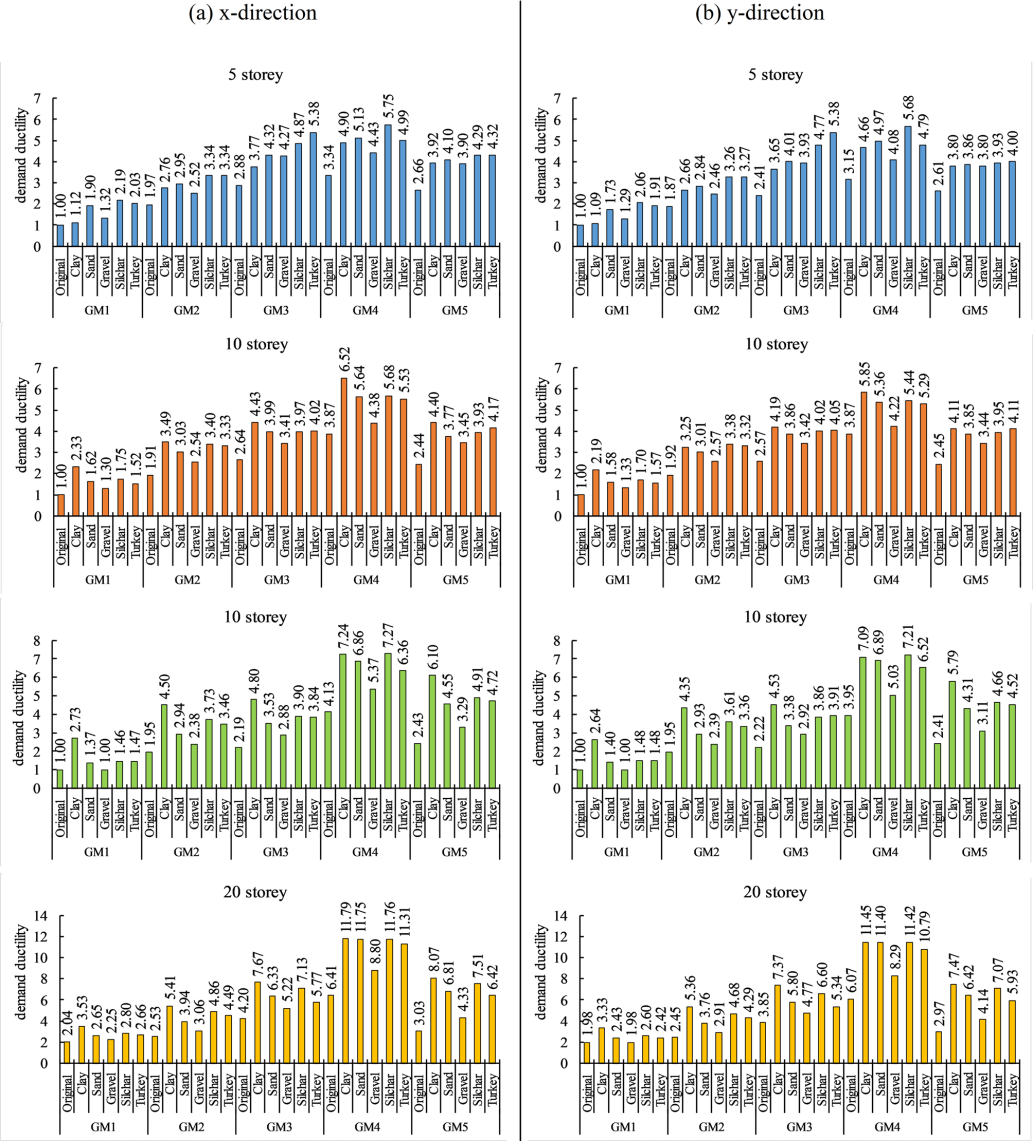
Demand ductility variations due to bedrock and surface-level ground motions.

Demand ductility is calculated as the ratio of the maximum roof displacement (from time history analysis) to the yield displacement obtained from the pushover curve for each case. Figure 13 represents the soil amplification effect on demand ductility of the building of various heights for different soil profiles and ground motions. As the ground motion intensity increases, the soil amplification effects also increase. The results indicate that due to soil amplification, the demand ductility can increase from 0.12 to a maximum of 2.88 for far-field ground motions, and from 0.51 to a maximum of 5.38 for near-field ground motions. In near-field ground motions, the presence of velocity pulses and strong directivity effects leads to significantly higher peak roof displacements compared to far-field motions. This behavior results in a wider and higher range of demand ductility values. The purpose of presenting these ranges is to simplify the interpretation of results and highlight the influence of soil amplification and ground motion characteristics on structural response. Thus, near-field ground motion increased the ductility demand by 40–86% more as compared to far-field ground motions. Various soil profiles also increase the demand for ductility in the building. It has been observed that the ductility demand of the building increased by up to 40–173% for clay, 51–125% for sand, 21–48% for gravel, 61–148% for Silchar, and 71–112% for the Turkey soil profile. For low-rise buildings (5 storeys), the layered soil profiles (Silchar and Turkey) resulted in the maximum increase (103–119%) in demand ductility compared to clay (47%) and sand (90%). On the other hand, for mid to high-rise buildings (10 to 20 storey), clay soil caused the maximum increase (114–173%) compared to the layered soil profiles (61–148%). Purely Gravel soil profile shows the minimum variation in ductility demand of the building (21–48%) in all cases. Thus, for low-rise buildings, the layered soil profiles are more vulnerable than purely clay, sand, or gravel. However, for high-rise buildings, purely clay soil is more vulnerable compared to the layered soil profiles. Additionally, high-rise buildings show higher increments in ductility demand than low- and mid-rise buildings. It was seen that the demand ductility had been increased by 32–119% for 5 storeys, 33–133% for 10 storeys, 22–173% for 15 storeys, and 21–166% for 20-storey buildings. x directions demand ductility are higher than the y direction demand ductility of the building.

### Fragility analysis

**Fig. 14:**
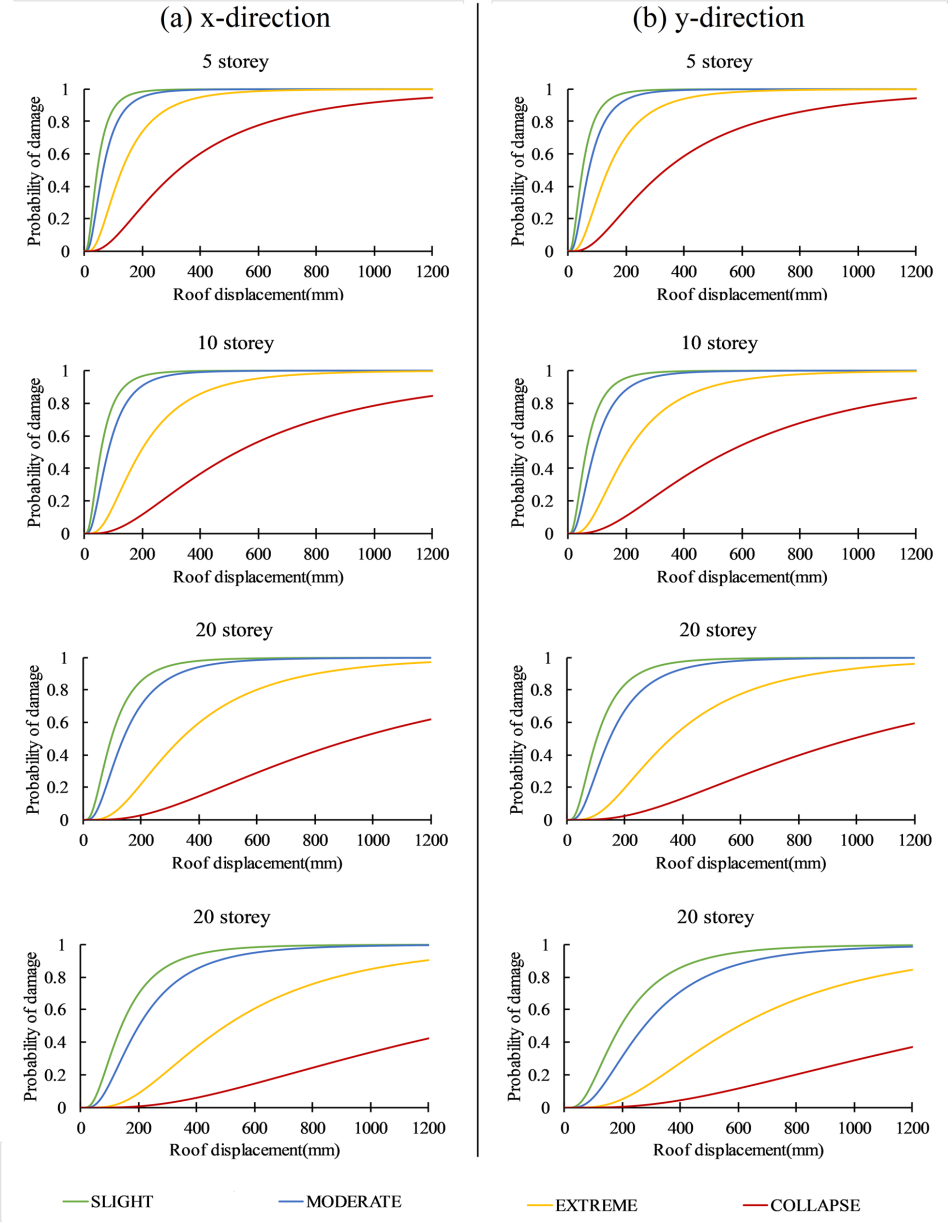
Fragility curves depicting seismic vulnerability in x and y directions.

The fragility analysis of the building can help estimate its seismic vulnerability in terms of the probability of damage, damage index, and recovery time. The fragility curves of 5,10,15 and 20-story buildings in various damage states are presented in Fig. 14. The fragility curve represents the cumulative probability of damage on the y-axis and spectral displacement on the x-axis. The probability of slight, moderate, extreme, and collapse damage can be estimated from the fragility curve for the particular seismic response (spectral displacement) of the building. It can be seen that fragility curves are nonlinear in nature, and the probability of damage in different damage states increases as the spectral displacement value increases. Tables 7 and 8 show the probability of damage to the building in the x and y direction for bedrock level GM and surface level (amplified) ground motion. The probability of damage to the building has increased in all ground motion due to the soil amplification effect.

The probability of damage in near-field ground motion was observed more than in far-field ground motion. The results show that in the far field ground motion, the probability of collapse damage increased by up to 12.5% in 5-storey, 9.7% in 10-storey, 6.75% in 15 storey, 2.37% in 20 storey buildings, and in near field ground motion, probability of collapse damage increased by up to 37.2% in 5 storey, 24.7% in 10 storey, 24% in 15 storey, and 25% in 20 storey building. Thus, near-field ground motion can amplify the collapse damage 1.5–10 times as compared to far-field ground motion, and low-rise buildings are more vulnerable (up to 13% more) than mid to high-rise buildings. Similarly, the different soil profiles affect the collapse damage differently. The result shows that the collapse damage of the buildings can be increased by up to 25.4% in clay, 28.8% in sand, 17.7% in gravel, 36.8% in Silchar, and 37.2% in Turkey. Similarly, the different building heights are greatly influenced by the type of soil used. For example, for low-rise buildings, the Silchar and turkey soil profile can increase the collapse damage by up to 37.2% as compared to the clay (25.4%) and sand (28.8%). In mid-to high-rise buildings, the predominant soil is clay, which increases the collapse damage by up to 25% compared to Silchar (22%) and Turkey soil profile (16.5%). The gravel soil shows the minimum increase in collapse damage (up to 17.7%) as compared to the other soil profile. Thus, the layered soil profile is more vulnerable for low-rise buildings, and the Clay soil profile is more vulnerable for mid to high rise building.

**Table 7:** Cumulative probability of damage (in %) to the Building in different damage States in the x-direction.

Soil	GM	5 storey				10 storey				15 storey				20 storey			
		Sli.	Mod.	Ext.	Coll.	Sli.	Mod.	Ext.	Coll.	Sli.	Mod.	Ext.	Coll.	Sli.	Mod.	Ext.	Coll.
GM1	Original	8.9	3.0	0.2	0.0	16.2	6.3	0.3	0.0	3.7	0.9	0.0	0.0	8.2	2.6	0.1	0
	Clay	22.8	10.0	1.2	0.1	84.3	68.8	22.1	3.0	73.2	53.8	11.2	1.1	61.6	40.8	5.7	0.5
	Sand	55.0	34.3	8.0	1.1	54.8	34.2	4.9	0.4	12.9	4.6	0.1	0.0	28	13	0.7	0
	Gravel	30.4	14.7	2.1	0.2	31.4	15.3	1.2	0.1	5.6	1.6	0.0	0.0	15.2	5.8	0.2	0
	Silchar	67.0	46.6	13.9	2.1	61.7	40.9	7.0	0.7	17.3	6.9	0.3	0.0	34.9	17.7	1.2	0.1
	Turkey	61.1	40.3	10.6	1.5	49.4	29.2	3.7	0.3	17.9	7.2	0.3	0.0	28.4	13.3	0.7	0
GM2	Original	58.6	37.8	9.5	1.3	69.2	49.1	10.2	1.1	41.4	22.6	2.0	0.1	23.2	10.1	0.5	0
	Clay	87.6	73.9	35.4	7.5	96.2	89.8	50.0	10.8	94.2	85.6	39.4	6.9	87.8	74.2	23.8	3.1
	Sand	90.2	78.2	40.6	9.3	93.6	84.4	39.9	7.4	77.9	59.8	14.3	1.6	70.6	50.7	9.1	0.8
	Gravel	83.2	67.1	28.5	5.5	88.3	75.0	27.9	4.2	62.3	41.5	6.4	0.6	47	27.1	2.5	0.2
	Silchar	94.2	85.6	51.7	13.8	95.7	88.7	47.7	10.0	89.0	76.1	27.0	3.8	83.1	67.1	17.9	2
	Turkey	94.2	85.6	51.7	13.8	95.4	88.0	46.4	9.5	86.2	71.6	22.6	2.9	79	61.2	14.2	1.5
GM3	Original	89.4	76.8	38.9	8.7	89.7	77.3	30.4	4.8	53.2	32.6	4.0	0.3	74.9	55.9	11.4	1.1
	Clay	96.5	90.3	60.9	18.6	99.1	96.9	72.1	22.9	95.4	88.0	43.9	8.3	96.5	90.4	47.3	9.2
	Sand	98.4	95.1	73.3	27.4	98.5	95.2	65.1	18.2	86.9	72.8	23.7	3.2	92.8	82.8	33.7	5.2
	Gravel	98.0	94.0	70.2	24.8	95.8	88.8	47.9	10.1	76.8	58.3	13.4	1.4	86.3	71.9	21.7	2.7
	Silchar	99.4	97.9	84.1	38.7	98.4	95.1	64.8	18.0	90.5	78.7	29.9	4.4	95.3	87.9	42.1	7.5
	Turkey	99.7	98.8	88.8	45.9	98.5	95.3	65.7	18.5	90.0	77.8	28.8	4.2	90.1	78	27.7	3.8
GM4	Original	94.2	85.6	51.7	13.8	98.2	94.5	62.6	16.8	92.1	81.7	33.6	5.3	93.1	83.4	34.5	5.4
	Clay	99.4	98.0	84.4	39.2	99.9	99.4	88.8	41.5	99.6	98.6	80.0	29.1	99.7	99	82.2	30.9
	Sand	99.6	98.4	86.8	42.6	99.7	98.8	83.8	34.2	99.5	98.0	76.3	25.5	99.6	98.5	78.5	27.1
	Gravel	98.9	96.4	77.8	31.5	99.0	96.7	71.4	22.4	97.1	91.7	52.1	11.2	98	94.1	57.3	13.1
	Silchar	99.8	99.2	91.3	50.6	99.7	98.9	84.0	34.5	99.6	98.6	80.2	29.3	99.7	98.9	81.3	29.9
	Turkey	99.5	98.2	85.4	40.5	99.7	98.7	83.0	33.2	99.0	96.7	69.0	20.0	99.4	97.9	74.3	23.4
GM5	Original	86.0	71.4	32.7	6.7	86.7	72.4	25.3	3.7	64.2	43.5	7.0	0.6	45.9	26.2	2.4	0.2
	Clay	97.0	91.5	63.7	20.3	99.1	96.8	71.7	22.6	98.3	94.9	61.5	15.6	97.1	91.9	50.9	10.5
	Sand	97.5	92.8	66.7	22.3	97.9	93.7	60.2	15.5	94.4	85.9	40.1	7.1	94.4	86.1	38.8	6.5
	Gravel	96.9	91.4	63.4	20.1	96.0	89.3	49.0	10.5	84.0	68.3	19.9	2.5	76.8	58.3	12.6	1.3
	Silchar	98.2	94.5	71.6	26.0	98.4	94.9	64.1	17.6	95.8	88.9	45.6	8.8	96.2	89.7	45.7	8.6
	Turkey	98.5	95.1	73.5	27.5	98.8	96.0	68.3	20.2	95.1	87.4	42.7	7.9	93.1	83.5	34.6	5.4

**Table 8:** Cumulative probability of damage (in %) to the Building in different damage States in the y-direction.

Soil	GM	5 storey				10 storey				15 storey				20 storey			
		Sli.	Mod.	Ext.	Coll.	Sli.	Mod.	Ext.	Coll.	Sli.	Mod.	Ext.	Coll.	Sli.	Mod.	Ext.	Coll.
GM1	Original	7.0	2.1	0.2	0.0	12.1	4.3	0.2	0.0	2.8	0.7	0.0	0.0	5.2	1.5	0.0	0.0
	Clay	18.1	7.3	0.9	0.1	77.7	59.5	16.8	2.2	69.1	49.0	9.2	0.9	57.8	37.0	4.9	0.4
	Sand	46.9	27.1	6.1	0.8	48.4	28.3	3.9	0.4	11.2	3.9	0.1	0.0	22.3	9.6	0.4	0.0
	Gravel	25.4	11.5	1.7	0.2	26.3	12.0	0.9	0.1	4.2	1.1	0.0	0.0	10.6	3.6	0.1	0.0
	Silchar	60.3	39.5	11.3	1.8	55.1	34.4	5.5	0.5	15.5	5.9	0.2	0.0	28.8	13.6	0.8	0.1
	Turkey	54.6	33.9	8.7	1.3	47.6	27.7	3.7	0.3	16.0	6.2	0.2	0.0	21.8	9.3	0.4	0.0
GM2	Original	52.7	32.2	8.1	1.2	66.2	45.7	9.6	1.0	39.6	21.2	1.8	0.1	23.1	10.1	0.5	0.0
	Clay	83.8	68.0	31.4	6.7	93.7	84.7	42.4	8.5	92.7	82.6	35.4	5.8	87.8	74.2	24.5	3.3
	Sand	86.8	72.6	36.1	8.2	92.0	81.4	37.5	6.9	76.3	57.7	13.3	1.4	68.2	47.9	8.4	0.8
	Gravel	79.2	61.6	25.6	5.0	86.6	72.3	26.8	4.2	61.2	40.4	6.2	0.5	43.7	24.4	2.2	0.2
	Silchar	92.0	81.5	47.4	12.6	94.5	86.3	45.1	9.4	86.6	72.3	23.7	3.2	81.7	65.0	17.1	2.0
	Turkey	92.1	81.7	47.8	12.7	94.2	85.7	44.0	9.0	83.6	67.7	19.9	2.5	76.9	58.5	13.2	1.4
GM3	Original	77.6	59.4	23.8	4.5	86.6	72.3	26.8	4.2	53.3	32.7	4.1	0.3	69.9	49.9	9.1	0.9
	Clay	94.9	87.0	56.5	17.0	98.4	94.9	66.1	19.4	93.6	84.5	38.1	6.6	96.0	89.3	45.9	8.8
	Sand	97.7	93.3	70.3	25.9	97.2	92.1	57.6	14.6	83.9	68.2	20.2	2.5	90.5	78.8	29.3	4.3
	Gravel	97.3	92.4	67.8	24.0	94.7	86.7	45.9	9.7	76.0	57.4	13.2	1.4	82.7	66.4	18.1	2.1
	Silchar	99.1	97.0	81.9	37.1	97.9	93.8	62.4	17.2	89.1	76.4	27.6	4.0	93.9	85.1	38.0	6.4
	Turkey	99.5	98.3	87.3	44.5	98.0	94.0	63.1	17.5	89.6	77.2	28.5	4.2	87.6	73.9	24.2	3.2
GM4	Original	90.9	79.4	44.5	11.3	97.3	92.3	58.0	14.8	89.9	77.8	29.2	4.3	91.9	81.2	32.3	4.9
	Clay	99.0	96.7	80.6	35.6	99.7	98.7	84.1	35.4	99.4	97.9	75.9	25.3	99.7	98.7	80.9	29.8
	Sand	99.3	97.5	83.9	39.7	99.5	98.2	80.4	31.1	99.3	97.4	73.0	23.0	99.5	98.2	76.8	25.7
	Gravel	98.0	94.0	72.0	27.3	98.5	95.1	66.8	19.8	95.7	88.6	45.6	8.9	97.5	92.8	54.3	12.0
	Silchar	99.7	98.7	89.3	48.1	99.5	98.3	81.1	31.8	99.5	98.1	77.1	26.4	99.6	98.6	79.8	28.6
	Turkey	99.2	97.1	82.1	37.4	99.5	98.1	79.9	30.5	98.6	95.5	64.5	17.3	99.3	97.5	72.3	22.2
GM5	Original	82.8	66.6	30.0	6.3	84.4	68.9	23.8	3.5	62.0	41.2	6.4	0.6	46.0	26.3	2.5	0.2
	Clay	95.7	88.7	59.7	18.7	98.2	94.4	64.4	18.3	97.6	92.9	55.7	12.8	96.2	89.8	46.8	9.1
	Sand	96.3	90.1	62.5	20.5	97.2	92.0	57.3	14.5	92.4	82.2	34.7	5.7	93.3	83.8	36.0	5.9
	Gravel	95.7	88.7	59.7	18.7	94.9	87.0	46.4	9.9	79.6	62.1	16.0	1.8	74.8	55.7	11.7	1.2
	Silchar	97.3	92.4	67.8	24.0	97.7	93.2	60.7	16.2	94.2	85.6	40.0	7.1	95.3	87.9	42.9	7.8
	Turkey	97.7	93.3	70.1	25.7	98.2	94.5	64.6	18.4	93.6	84.4	38.0	6.5	91.2	80.0	30.8	4.6

### Damage index and recovery time

The DI (Damage Index) value has significantly increased for all ground motions and different soil models due to the soil amplification effect. Table 9 represents the DI values of the 5, 10, 15, and 20-storey buildings for original and amplified ground motion due to different soil profiles. For far-field ground motions, the DI value increased by 0.02 to 1.6, with a maximum DI value of 2.45. In contrast, for near-field ground motions, the DI value increased by 0.18 to 1.81, reaching a maximum DI value of 3.41. As a result, buildings subjected to near-field ground motions experienced moderate to severe damage states, while those subjected to far-field ground motions experienced slight to moderate damage. It is important to note that for near-field ground motions, even a slight increase in the DI value leads to more severe damage compared to far-field ground motions. Hence, for most severe ground motion (GM 3 and GM 4), the DI value increased by up to 31–102% for clay, 37–108% for sand, 20–67% for gravel, 37–127% for Silchar, and 38–123% for the Turkey soil profile. For low-rise buildings (5 storey), the combination of soil profiles (Silchar and Turkey) resulted in the maximum increase (up to 56%) in damage index compared to clay (31%) and sand (37%). In contrast, for mid to high-rise buildings (10 to 20 storey), clay soil caused the maximum increase (up to 44–162%) compared to the combination of soil profiles (up to 38–123%). For near-field ground motion, the recovery time of the building has increased up to 383 days as compared to the 268 days for far-field ground motion, as presented in Table 9. Similarly, the various soil profiles also increase the building’s recovery time. It has been observed that the recovery time of the building has increased by up to 327 days in clay, 282 days in sand, 210 days in gravel, 335 days in silchar, and 383 days in turkey soil profile. For various heights of the building, the predominant soil is different. For example, for a low-rise (5-storey) building, the maximum increase in recovery time is observed in layered soil (silchar and turkey) up to 335–383 days as compared to the clay (254 days) and sand (279 days). In contrast, for mid to high-rise buildings, a maximum increase in recovery time is observed for clay soil up to 286–327 days as compared to the Silchar (232–308 days) and turkey soil (215–261 days) profile. Increase in Recovery time of the building due to soil amplification observed maximum for low rise buildings (up to 383 days in 5 storey) for the mid-rise building is (up to 286 days for 10 storey) which has increased for high rise building up to 316–327 days for 15–20 storey due to the torsional effect. The torsional effects with building height increases due to their greater height and flexibility. This results in larger displacements and more significant damage, further prolonging recovery.

**Table 9:** Soil amplification effects on damage index and estimated recovery time of buildings.

GM	Soil	Damage Index								Recovery time (days)							
		x-direction				y-direction				x-direction				y-direction			
		5 st.	10 st.	15 st.	20 st.	5 st.	10 st.	15 st.	20 st.	5 st.	10 st.	15 st.	20 st.	5 st.	10 st.	15 st.	20 st.
GM 1	Original	0.12	0.23	0.05	0.05	0.09	0.17	0.03	0.02	5	10	1	1	4	7	1	1
	Clay	0.34	1.78	1.39	0.76	0.26	1.56	1.28	0.65	18	178	112	48	14	144	98	39
	Sand	0.99	0.94	0.18	0.23	0.81	0.81	0.15	0.16	77	63	7	10	61	52	6	6
	Gravel	0.47	0.48	0.07	0.10	0.39	0.39	0.05	0.06	28	25	2	4	22	20	2	2
	Silchar	1.30	1.10	0.24	0.31	1.13	0.95	0.22	0.22	118	80	10	15	99	66	9	10
	Turkey	1.14	0.83	0.25	0.23	0.98	0.79	0.22	0.15	96	52	11	10	80	50	9	6
GM 2	Original	1.07	1.30	0.66	0.18	0.94	1.23	0.63	0.17	88	103	37	7	75	97	35	7
	Clay	2.04	2.47	2.26	1.57	1.90	2.29	2.16	1.54	254	340	279	148	228	296	256	145
	Sand	2.18	2.25	1.54	0.97	2.04	2.18	1.49	0.88	286	281	132	69	258	267	126	60
	Gravel	1.84	1.95	1.11	0.49	1.71	1.90	1.08	0.42	211	212	78	26	192	205	75	21
	Silchar	2.45	2.42	1.96	1.37	2.33	2.35	1.86	1.28	356	327	208	117	330	312	189	107
	Turkey	2.45	2.39	1.83	1.22	2.34	2.33	1.74	1.12	356	319	183	97	332	305	166	86
GM 3	Original	2.14	2.02	0.90	1.09	1.65	1.90	0.90	0.92	275	227	57	82	181	205	57	64
	Clay	2.66	2.91	2.36	2.20	2.55	2.79	2.23	2.12	418	486	304	275	390	445	271	258
	Sand	2.94	2.77	1.87	1.86	2.87	2.62	1.75	1.69	510	436	189	200	490	389	168	170
	Gravel	2.87	2.43	1.50	1.50	2.81	2.37	1.48	1.32	485	328	127	137	470	316	125	112
	Silchar	3.20	2.76	2.04	2.07	3.15	2.71	1.97	1.93	606	434	225	245	589	420	212	215
	Turkey	3.33	2.78	2.01	1.69	3.30	2.73	1.99	1.53	658	440	218	169	646	425	216	144
GM 4	Original	2.45	2.72	2.13	1.88	2.26	2.62	2.01	1.77	356	420	246	205	311	391	220	185
	Clay	3.21	3.30	3.07	2.99	3.12	3.18	2.99	2.94	610	638	546	521	577	591	513	500
	Sand	3.27	3.17	2.99	2.90	3.20	3.09	2.93	2.83	635	584	515	487	610	557	490	463
	Gravel	3.05	2.90	2.52	2.42	2.91	2.80	2.39	2.32	547	481	352	334	503	450	314	307
	Silchar	3.41	3.17	3.08	2.97	3.36	3.11	3.01	2.91	691	587	548	513	671	562	522	490
	Turkey	3.23	3.15	2.85	2.80	3.16	3.08	2.76	2.73	619	577	461	452	592	552	430	428
GM 5	Original	1.97	1.88	1.15	0.47	1.86	1.81	1.10	0.45	237	197	83	25	220	187	77	24
	Clay	2.72	2.90	2.70	2.28	2.63	2.75	2.59	2.14	437	483	410	296	412	433	374	263
	Sand	2.79	2.67	2.27	1.99	2.69	2.61	2.15	1.88	459	404	282	227	432	386	252	205
	Gravel	2.72	2.45	1.75	1.15	2.63	2.38	1.60	1.05	435	334	167	88	412	319	143	79
	Silchar	2.90	2.75	2.39	2.16	2.81	2.68	2.27	2.05	496	429	314	266	470	409	282	241
	Turkey	2.95	2.83	2.33	1.89	2.87	2.76	2.22	1.73	511	458	297	205	488	434	270	177

### Seismic vulnerability index

**Fig. 15:**
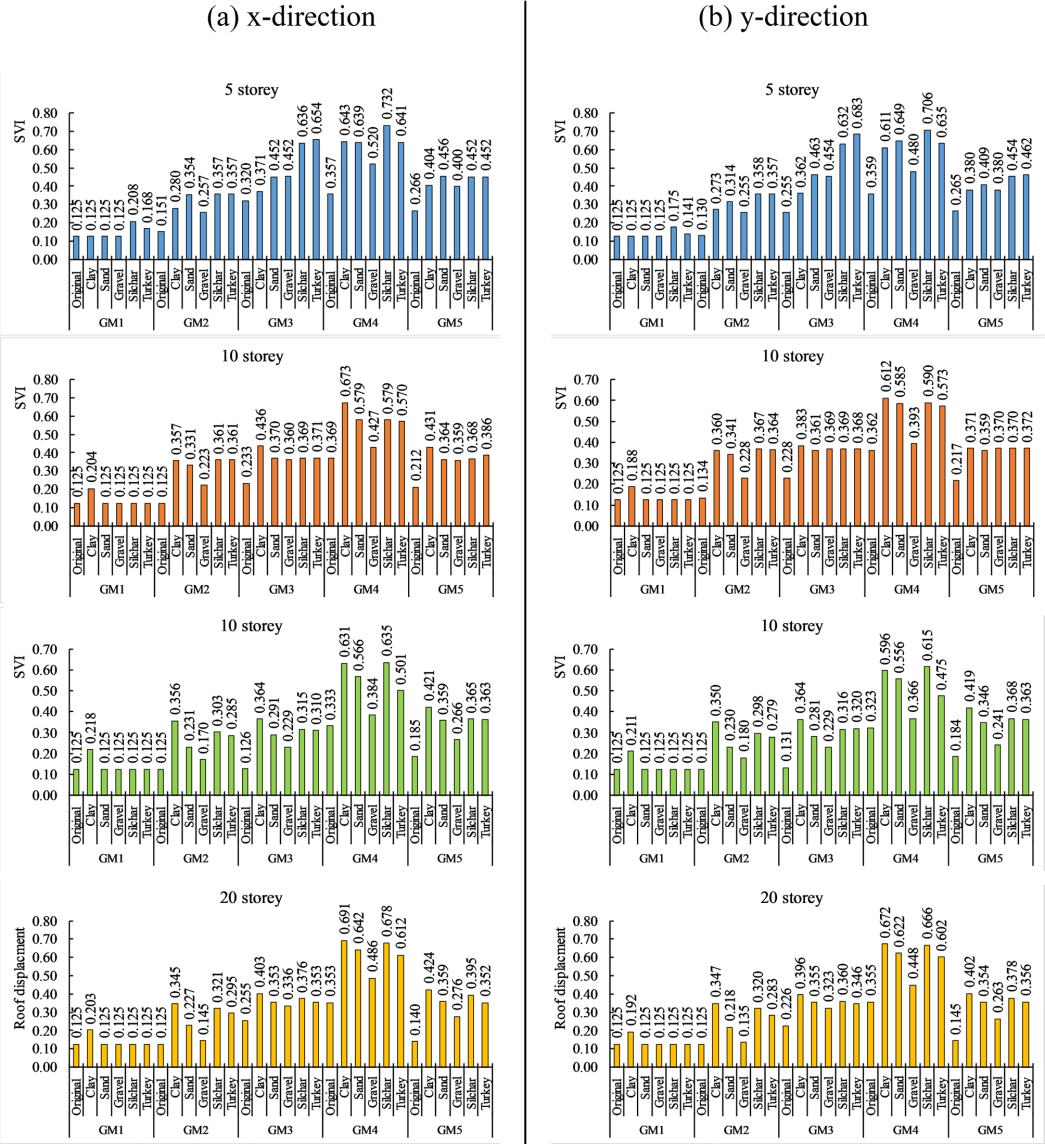
Seismic vulnerability index for the building in x and y directions.

Seismic vulnerability is also one of the important parameters for knowing the physical condition of the building after a particular seismic activity. From the pushover analysis, the structural performance of a building can be evaluated at different performance levels, such as Immediate Occupancy (IO), Life Safety (LS), and Collapse Prevention (CP). Also, it provides information regarding the formation of plastic hinges in the various structural members of the building. By knowing the number of plastic hinge formations in different beams and columns, the seismic vulnerability index (SVI) can be estimated. The plastic hinge formation in the 5, 10, 15, and 20-story buildings at different performance levels for various ground motions and soil profiles are presented in Table 10 in the x direction and Table 11 in the y direction. The results show that the number of plastic hinge formations has increased for amplified ground motion in all building cases. Additionally, for near-field ground motion, a greater number of plastic hinges move to higher damage states compared to far-field ground motion. For example, in a 20-storey building on clay soil under GM 4, 288 hinges shift from the IO-LS state to 137 hinges in the LS-CP state and 162 hinges in the CP < state. In contrast, under GM 2, 172 hinges in the B-IO state shift to 260 hinges in the IO-LS state. This demonstrates the severity of the impact of soil amplification on near-field ground motion compared to far-field ground motion. Similarly, different soil profiles increase the number of plastic hinge formations or cause them to move to higher damage states. For instance, under GM 4 in a 5-storey building, the structure is in the IO-LS state (80 hinges) for bedrock conditions (original). However, this shifts to higher damage states, such as the CP < state, with 18 hinges for clay, 36 hinges for sand, 4 hinges for gravel, 71 hinges for Silchar, and 27 hinges for Turkey soil profiles. The formation of plastic hinges and their progression to higher damage states increase for high-rise buildings under near-field ground motion. The above result can be understood better by knowing how the Seismic vulnerability index changes with the soil amplification effect. Figure 15 shows the Seismic vulnerability index of the 5, 10, 15, and 20-storey buildings for various soil profiles and ground motions. The increment in SVI value was observed more for near-field ground motion as compared to the far-field ground motion. For instance, SVI values are increased by up to + 0.236, showing a maximum SVI value of 0.361 (moderate damage) for far-field ground motion. On the other hand, the SVI value increased by up to + 0.375, showing a maximum SVI value of 0.732 (severe to collapse) for near-field ground motion. Thus, for near-field ground motion, a small increment in SVI value can lead to severe damage to the building as compared to the far-field ground motion. Similarly, different soil profile amplifies the SVI value, such as up to 80–179% in clay, 58–165% in sand, 16–82% in gravel, 58–189% in silchar, and 59–189% in turkey soil profile. For near-field ground motion, low-rise buildings (5 storey), the layered soil profiles (Silchar and Turkey) resulted in the maximum increase (up to 105%) in SVI value compared to clay (80%) and sand (79%). In contrast, for mid to high-rise buildings (10–20 storey), clay soil caused the maximum increase (87–189%) compared to the layered soil profiles (59–146%). Soil amplification also affects the different heights of the building since it has been observed that, for near field ground motion, the SVI value increased by up to 46–105% in 5 storey, 54–87% in 10 storey, 82–189% in 15 storey and 38–106% in 20 storey building.

**Table 10:** Impact of soil amplification on plastic hinge formation in buildings (x direction).

GM	Soil	5 storey				10 storey				15 storey				20 storey			
		B-IO	IO-LS	LS-CP	CP<	B-IO	IO-LS	LS-CP	CP<	B-IO	IO-LS	LS-CP	CP<	B-IO	IO-LS	LS-CP	CP<
GM 1	Original	1	0	0	0	1	0	0	0	1	0	0	0	113	0	0	0
	Clay	7	0	0	0	124	40	0	0	158	63	0	0	248	78	0	0
	Sand	43	0	0	0	74	0	0	0	49	0	0	0	208	0	0	0
	Gravel	16	0	0	0	30	0	0	0	2	0	0	0	138	0	0	0
	Silchar	40	14	0	0	85	0	0	0	62	0	0	0	247	0	0	0
	Turkey	42	6	0	0	68	0	0	0	46	0	0	0	212	0	0	0
GM 2	Original	42	4	0	0	114	0	0	0	127	0	0	0	170	0	0	0
	Clay	44	40	0	0	57	142	0	0	84	208	0	0	124	260	0	0
	Sand	29	69	0	0	64	112	0	0	158	77	0	0	232	107	0	0
	Gravel	48	32	0	0	121	52	0	0	174	28	0	0	271	18	0	0
	Silchar	32	80	0	0	52	137	0	0	126	152	0	0	146	221	0	0
	Turkey	32	80	0	0	51	134	0	0	134	130	0	0	169	186	0	0
GM 3	Original	37	55	0	0	117	59	0	0	173	1	0	0	208	138	0	0
	Clay	30	78	3	0	36	163	26	0	79	221	0	0	95	300	33	0
	Sand	33	68	32	1	55	166	0	0	131	136	0	0	122	288	0	0
	Gravel	31	65	32	0	52	137	0	0	157	74	0	0	133	246	0	0
	Silchar	17	51	57	25	55	166	0	0	117	165	0	0	96	320	1	0
	Turkey	19	44	56	33	54	167	0	0	121	160	0	0	118	278	0	0
GM 4	Original	32	80	0	0	55	165	0	0	102	181	0	0	123	288	0	0
	Clay	17	49	56	28	24	44	145	26	13	147	169	24	50	148	131	173
	Sand	20	49	52	32	32	81	127	0	34	159	160	0	74	137	158	121
	Gravel	29	53	56	0	37	166	22	0	70	229	8	0	80	234	129	0
	Silchar	20	27	42	64	32	81	127	0	12	145	170	25	59	145	137	163
	Turkey	19	49	54	30	34	84	123	0	50	196	105	0	60	154	201	55
GM 5	Original	46	35	0	0	125	46	0	0	168	38	0	0	272	13	0	0
	Clay	27	76	10	0	37	165	24	0	81	211	50	0	95	279	60	0
	Sand	21	72	23	0	58	161	0	0	83	211	0	0	116	298	0	0
	Gravel	27	76	9	0	55	140	0	0	145	111	0	0	188	162	0	0
	Silchar	32	66	33	0	56	165	0	0	79	224	0	0	93	310	21	0
	Turkey	33	68	34	0	48	171	4	0	80	219	0	0	124	288	0	0

**Table 11:** Impact of soil amplification on plastic hinge formation in buildings (y direction).

GM	Soil	5 storey				10 storey				15 storey					20 storey		
		B-IO	IO-LS	LS-CP	CP<	B-IO	IO-LS	LS-CP	CP<	B-IO	IO-LS	LS-CP	CP<	B-IO	IO-LS	LS-CP	CP<
GM 1	Original	0	0	0	0	1	0	0	0	0	0	0	0	93	0	0	0
	Clay	9	0	0	0	125	30	0	0	158	57	0	0	252	65	0	0
	Sand	40	0	0	0	70	0	0	0	48	0	0	0	170	0	0	0
	Gravel	26	0	0	0	25	0	0	0	2	0	0	0	126	0	0	0
	Silchar	45	8	0	0	79	0	0	0	62	0	0	0	216	0	0	0
	Turkey	45	2	0	0	70	0	0	0	45	0	0	0	167	0	0	0
GM 2	Original	45	1	0	0	110	3	0	0	132	0	0	0	176	0	0	0
	Clay	45	38	0	0	50	129	0	0	89	199	0	0	122	262	0	0
	Sand	38	53	0	0	59	117	0	0	163	78	0	0	242	97	0	0
	Gravel	48	32	0	0	118	55	0	0	170	34	0	0	275	9	0	0
	Silchar	32	80	0	0	46	135	0	0	127	145	0	0	145	217	0	0
	Turkey	32	80	0	0	48	133	0	0	137	123	0	0	180	169	0	0
GM 3	Original	48	32	0	0	118	55	0	0	183	4	0	0	235	105	0	0
	Clay	35	76	4	0	52	165	6	0	78	216	0	0	94	307	24	0
	Sand	33	68	32	4	60	158	0	0	136	125	0	0	116	281	0	0
	Gravel	37	64	32	4	45	137	0	0	163	77	0	0	143	224	0	0
	Silchar	14	52	51	26	55	166	0	0	116	165	0	0	115	299	0	0
	Turkey	15	40	53	40	56	166	0	0	113	169	0	0	122	261	0	0
GM 4	Original	31	80	0	0	59	160	0	0	110	172	0	0	118	287	0	0
	Clay	15	54	54	18	20	77	136	0	31	137	173	8	67	138	138	162
	Sand	20	44	53	36	19	96	114	0	45	149	159	0	72	143	183	87
	Gravel	28	71	34	4	51	161	11	0	79	225	0	0	89	260	87	0
	Silchar	23	37	25	71	20	92	118	0	22	139	171	17	70	137	138	157
	Turkey	15	51	51	27	20	101	107	0	60	198	87	0	64	144	219	36
GM 5	Original	46	35	0	0	122	48	0	0	168	37	0	0	271	18	0	0
	Clay	42	70	15	0	56	166	1	0	68	220	36	0	94	301	31	0
	Sand	41	69	23	1	61	157	0	0	92	194	0	0	121	290	0	0
	Gravel	42	70	15	0	45	138	0	0	162	89	0	0	199	147	0	0
	Silchar	37	64	32	4	54	165	0	0	76	223	0	0	97	318	4	0
	Turkey	34	67	32	4	55	166	2	0	78	215	0	0	116	284	0	0

### Limitation of the study

This study has a few limitations that should be acknowledged. The ground motion records used were originally recorded at the soil surface, but in this study, they were applied at the base of the soil model (i.e., at the foundation level of the structure). This approach does not fully capture the true site response, since the characteristics of surface-recorded motions can differ from those at deeper levels, such as bedrock or the base of the soil profile. As a result, the seismic demand on the structure may be affected. Additionally, the selected ground motions were not scaled to match the design response spectrum of IS 1893:2016, in order to retain their natural variability. While this helps reflect realistic seismic input, it may cause differences compared to code-based design expectations. The study used five ground motions, which is acceptable for an initial evaluation, but more records would improve the statistical strength of the results. Fragility curves were developed using pushover analysis, which may not fully capture the complex torsional behavior observed in the L-shaped building. Although time history analyses were also carried out, using more detailed nonlinear dynamic analyses could offer better insight into such effects. Lastly, soil–structure interaction and potential geotechnical issues like liquefaction were not considered, and future studies could include these to improve the accuracy and completeness of seismic assessments for irregular buildings.

## Conclusions

This study evaluates the seismic vulnerability of L-shaped unsymmetrical buildings with 5 to 20 storeys building under homogeneous and layered soil conditions. Five ground motions, which include two far-field and three near-field ground motions, are applied at the bedrock level, and amplification of ground motion has been observed at the surface level. Pushover and time history analysis have been performed to evaluate the capacity and seismic demand of the building for amplified ground motion such as roof displacement, ductility demand, and spectral displacement. A fragility analysis was performed to estimate the probability of damage, damage index, seismic vulnerability index, and recovery time of the building. Finally, the soil amplification effect on the seismic vulnerability parameter of different building heights is estimated due to various soil profiles and ground motions. This study shows the following conclusions:

- Soil amplification significantly increases ground motion intensity. Near-field motions show up to 320% increase in PGA and 633% in PSA, while soil profiles contribute up to 275% (PGA) and 390% (PSA) amplification. Clay soil exhibits maximum amplification for longer periods (1.5–4 s), while layered soils (Silchar, Turkey) are more critical for short periods (0–1.5 s).
- Roof displacement increases up to 211%, with clay soil affecting mid- to high-rise buildings most, and Silchar/Turkey soils dominating low-rise structures. Displacement increases with height: 190% (5-storey), 189% (10-storey), 211% (15-storey), 197% (20-storey).
- Near-field ground motions increase the probability of collapse damage by up to 20% compared to far-field motions. For low-rise buildings, maximum soil amplification occurs with near-field motions (GM4) and layered soils (Silchar/Turkey). For mid- to high-rise buildings, the highest amplification is from GM4 and clay soil. The least amplification occurs with far-field motions (GM1) and gravel soil, regardless of building height.
- Soil type also plays a critical role: Silchar and Turkey soils lead to up to 37.2% increase in collapse probability for low-rise buildings, while clay soil causes up to 25% for mid- to high-rise structures. Gravel soil shows the least impact (up to 17.7%). Low-rise buildings (5-storey) are the most vulnerable, experiencing the highest collapse rates, while taller buildings (10–20 storeys) show slightly lower but still significant damage levels (up to 24–25%), emphasizing the combined effect of soil type and building height on seismic vulnerability.
- Damage Index (DI) increases significantly under near-field ground motions, with up to 162% rise observed in clay soil for mid- to high-rise buildings and up to 127% in Silchar and Turkey soils for low-rise buildings.
- Recovery time extends considerably, reaching up to 383 days for low-rise buildings on layered soils and up to 327 days for mid-/high-rise buildings on clay soil.
- Seismic Vulnerability Index (SVI) rises by approximately 59% under near-field motions, with maximum increases of 189% for clay (mid-/high-rise) and up to 105% for layered soils (low-rise).
- Low-rise buildings are more vulnerable due to resonance with high-frequency content. As height increases, torsional effects become more prominent. Collapse damage varies from 37.2% (low-rise) to 24–29% (mid-/high-rise); recovery times from 383 to 327 days, and SVI from 105 to 189%.
- Vulnerability ranking varies by building height:
  - Low-rise: gravel < clay < sand < Silchar < Turkey.
  - Mid-/high-rise: gravel < sand < Silchar < Turkey < clay.
- While this research provides valuable insights into soil amplification effects on unsymmetrical buildings, certain limitations should be noted. The analysis used five surfaced ground motions to preserve their natural characteristics, though this limits statistical generalization. The surface-recorded motions applied at the model base may not fully represent site response. Pushover analysis was employed despite potential limitations in capturing complex torsional behavior in L-shaped structures and soil–structure interaction and liquefaction were not considered. Future research will address these by using bedrock-compatible ground motion inputs, incorporating SSI and liquefaction effects, and employing advanced dynamic analysis methods to enhance fragility assessments of irregular buildings, building on insights from Vicencio et al. (2024)^96^ on SSI in inelastic structures and Wang et al. (2025)^97^ on liquefaction impacts in reinforced concrete frame clusters.

The observed trends are primarily influenced by the dynamic interaction between building height and soil properties. Low-rise buildings, due to their shorter natural periods, resonate with the high-frequency seismic waves amplified by soft and layered soils, leading to higher seismic demands. In contrast, mid- to high-rise buildings, with longer natural periods, are more affected by the amplification of low-frequency waves, particularly in soft clay soils.

Layered soil profiles (like Silchar and Turkey), with varying stiffness and density, create complex wave propagation effects such as reflection and refraction, which intensify ground motion at specific frequencies—especially impacting low-rise buildings. On the other hand, homogeneous soils (clay, sand, gravel) show more uniform behavior, with clay being the most critical for taller buildings due to its low stiffness and high compressibility. Additionally, torsional effects in unsymmetrical (L-shaped) buildings contribute to increased damage, especially as height increases. The shift of plastic hinges to higher damage states under amplified surface motions further explains the elevated Damage Index and longer recovery times observed. These findings underscore the importance of site-specific soil conditions and building geometry in seismic design and performance evaluation.

This study holds significant importance for updating seismic design codes and standards, particularly for unsymmetrical L-shaped buildings, as it underscores the critical influence of soil amplification and near-field ground motions on structural damage. Current codes often overlook the specific effects of soil types. These findings suggest the inclusion of site-specific soil classification, height-dependent amplification factors, and distinct design spectra for near- and far-field motions in seismic codes. Additionally, the study recommends restricting construction on highly amplifying soils for high-rise unsymmetrical buildings in earthquake-prone regions.

## Data Availability

The datasets used and/or analysed during the current study available from the corresponding author on reasonable request.
